# Solid-state synthesis of ordered mesoporous carbon catalysts via a mechanochemical assembly through coordination cross-linking

**DOI:** 10.1038/ncomms15020

**Published:** 2017-04-28

**Authors:** Pengfei Zhang, Li Wang, Shize Yang, Jennifer A. Schott, Xiaofei Liu, Shannon M. Mahurin, Caili Huang, Yu Zhang, Pasquale F. Fulvio, Matthew F. Chisholm, Sheng Dai

**Affiliations:** 1 Chemical Sciences Division, Oak Ridge National Laboratory, Oak Ridge, Tennessee 37831, USA; 2 Key Laboratory for Advanced Materials and Research Institute of Industrial Catalysis, East China University of Science and Technology, Shanghai 200237, China; 3 Materials Science and Technology Division, Oak Ridge National Laboratory, Oak Ridge, Tennessee 37831, USA; 4 Department of Chemistry, University of Tennessee, Knoxville, Tennessee 37996, USA; 5 Neutron Science Directorate, Oak Ridge National Laboratory, Oak Ridge, Tennessee 37831, USA; 6 Department of Chemical and Biomolecular Engineering, Vanderbilt University, Nashville, Tennessee 37235, USA; 7 Department of Chemistry, University of Puerto Rico, Rio Piedras Campus, San Juan, Puerto Rico 00931, USA

## Abstract

Ordered mesoporous carbons (OMCs) have demonstrated great potential in catalysis, and as supercapacitors and adsorbents. Since the introduction of the organic–organic self-assembly approach in 2004/2005 until now, the direct synthesis of OMCs is still limited to the wet processing of phenol-formaldehyde polycondensation, which involves soluble toxic precursors, and acid or alkali catalysts, and requires multiple synthesis steps, thus restricting the widespread application of OMCs. Herein, we report a simple, general, scalable and sustainable solid-state synthesis of OMCs and nickel OMCs with uniform and tunable mesopores (∼4–10 nm), large pore volumes (up to 0.96 cm^3^ g^−1^) and high-surface areas exceeding 1,000 m^2^ g^−1^, based on a mechanochemical assembly between polyphenol-metal complexes and triblock co-polymers. Nickel nanoparticles (∼5.40 nm) confined in the cylindrical nanochannels show great thermal stability at 600 °C. Moreover, the nickel OMCs offer exceptional activity in the hydrogenation of bulky molecules (∼2 nm).

## Abstract (web summary)

Ordered mesoporous carbons have shown promise in a range of applications, but sustainable methods to achieve their large scale production are lacking. Here, Dai and coworkers produce OMCs via the mechanochemical assembly of non-toxic polyphenol-metal complexes and triblock copolymers, followed by pyrolysis.

Because of the uniform pore channel, large mesopore size, high pore volume and good chemical inertness, ordered mesoporous carbons (OMCs) have already demonstrated promising performance in a number of applications such as catalysis, separation, fuel cells and supercapacitors^1^,^2^,^3^,^4^,^5^. The wide and ordered pore channels are especially useful for the separation of large biomolecules or natural products and, at the same time, provide broad space for fast mass transfer, which is highly beneficial in high-rate supercapacitors and heterogeneous catalysis^6^,^7^,^8^. In general, two methods—a hard-templating route and a soft-templating strategy—have been used for the fabrication of OMCs. The hard templating technology usually starts with the synthesis of an ordered mesoporous silica, followed by inverse replication of the template using organic polymerization^9^,^10^,^11^. Since 1999—when the Ryoo group and the Hyeon group introduced OMCs for the first time—numerous OMCs with precisely controlled mesostructures have been synthesized^12^,^13^. However, the sacrificial nature of the ordered silica template together with specific requirement that HF, NH_4_HF_2_ or NaOH must be used to remove the template makes this process complicated, time consuming and costly^14^. In contrast, the soft templating strategy via a supramolecular self-assembly between amphiphilic nonionic co-polymers and phenolic resins actually provides a more feasible pathway to construct OMCs^15^,^16^,^17^. The so-called solvent evaporation induced self-assembly can lead to mesoscopic micelle-resol composites—driven by the hydrogen-bonding interaction (for example, PhOH···O). The triblock co-polymer as the soft template can subsequently decompose during further thermal treatment (for example, >400 °C), resulting in an ordered carbon mesostrucure^18^,^19^,^20^. Although the soft-templating method has significantly enhanced the chemistry of OMCs in the past decade, the key crosslinking reaction remains limited to the acid- or base-catalysed polycondensation of toxic phenol-formaldehyde using large amount of solvents. Moreover, relying exclusively on the chemistry of phenolic resin, which is composed of only carbon and oxygen neglects an opportunity to tune the OMC composition *in situ* rather than post-synthesis. Finally, the wet self-assembly method with multiple drying processes cannot be easily realized in the large-scale production of membranes^21^,^22^,^23^,^24^. Thus, overcoming the drawbacks of both the hard-templating and soft-templating methods is extremely important for advancing and commercializing OMC-based technologies.

To date, the development of a facile assembly for OMC production is still highly challenging because several rigorous criteria—including strong electrostatic interactions with mesophase micelles, presence of reactive groups for polymerization and crosslinking, organized polymerization around micelle aggregates, avoidance of polymerization-induced phase separation, rigid polymer backbones to prevent mesopore collapse and ability to form carbon frameworks—must be simultaneously satisfied^25^,^26^,^27^,^28^,^29^,^30^. In addition, the applicability of an OMC support could be tremendously enhanced if metal nanoparticles (NPs) could be *in situ* confined inside the pores to create a dream catalyst—restricting the growth and aggregation of metal NPs, minimizing metal loss and enabling even large reactants to undergo rapid mass transfer during heterogeneous catalysis^31^,^32^. Actually, several modifications to the soft-templating approach using metal-salt additives can result in metal NP-OMC hybrids, but the NP size is much larger than the pore size of the OMC—in other words, the NPs are not confined in the mesoporous channels^33^,^34^,^35^. The lack of suitable interactions between metal salts and phenol-formaldehyde precursors may lead to the uncontrolled growth of NPs.

In this contribution, we demonstrate a versatile mechanochemical self-assembly approach to fabricate polyphenols metal (Zn^2+^ or Ni^2+^) and poly(ethylene oxide)–poly(propylene oxide)–poly(ethylene oxide) (PEO–PPO–PEO) mesophase composites by ball milling, which offers an easy synthesis of OMCs after pyrolysis treatment. The resultant carbon materials possess a hexagonally ordered mesostructure with a narrow pore size distribution (∼7–8 nm), high-surface area (up to 1,057 m^2^ g^−1^), and large pore volume (up to 0.96 cm^3^ g^−1^). In the case of the Ni-based coordination crosslinking, the reduction of ligand-stabilized Ni^2+^ ions could occur around micelles during carbonization, therefore, inherently endowing OMCs with small (∼4.35 nm), evenly dispersed and high content (∼16.1% wt) Ni NPs. In addition, Ni NPs confined in the mesoporous channel and carbon matrix can resist thermal treatment even at 600 °C, which is rarely achieved in carbon-supported metal catalysts. Importantly, our Ni-OMC shows an exceptional activity toward the selective hydrogenation of large biomolecules (for example, cholesteryl acetate: ∼1.87 nm), a key process for hormone medicines that is not accessible by Ni supported on activated carbon. Compared with wet phenol-formaldehyde polycondensation, the essence of mechanochemical coordination polymerization relies on three features: (1) the coordination polymerization can be completed by ball milling, or in other words, the solvent-free crosslinking is restricted in a specific space—void of mesophase micelles; (2) the metal-salt linkers can *in situ* become stable metal NPs within the pores of the OMC; (3) the solid-state polymerization can exploit monomers with no or poor solubility in solvents, a vital issue that often makes solution self-assembly fail.

## Results

### Fabrication of OMCs

**Figure 1:**
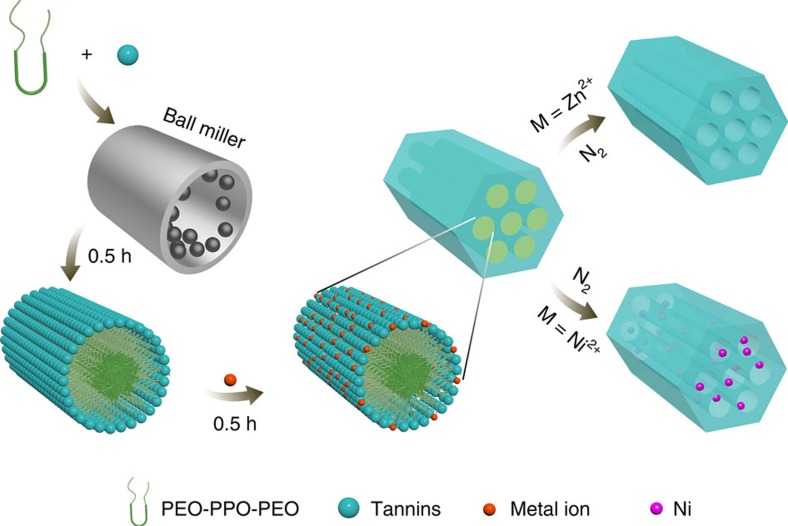
Synthesis of OMCs. A proposed mechanism for the mechanochemical assembly mediated by coordination crosslinking of tannin, a biomass-derived polyphenol, with divalent metal ions in the presence of Pluronic triblock co-polymers PEO–PPO–PEO. First, the PEO–PPO–PEO and tannin are ball milled for 0.5 h, forming a brown gel. Selected metal acetates are then added to the miller, resulting in homogeneous gel nanocomposites after a short milling time (0.5 h). After carbonization in a N_2_ atmosphere, pure OMCs, or metal NP OMCs are obtained depending on the boiling point of the reduced metal species. For example, metallic Zn can evaporate during high-temperature treatment.

Figure 1 describes a simple route based on the mechanochemical self-assembly of tannin, metal salts and PEO–PPO–PEO. Tannin is a renewable and inexpensive polyphenol with a molecular weight ranging from 500 to 3,500 (world production: ∼220,000 ton per year), and inherent pyrogallol- and catechol-like sites on tannin that can in principle function for both hydrogen-bonding and coordination interactions (Supplementary Fig. 1). The self-assembly behaviour between tannin and an amphiphilic surfactant in solution conditions has already been confirmed by pioneering work^36^,^37^,^38^,^39^. In our current study, the ability of tannin to form hydrogen-bonding networks with F127 in the solid state was explored. Tannin (1 g) and F127 (1 g) were mechanically ground in a vibrating ball miller for 30 min. It is important to note that no solvents were used in this process. Compared with tannin, the –OH sorption peak in the tannin-F127 mixture demonstrates a clear red shift in the Fourier transform infrared spectrum, revealing the formation of a possible hydrogen-bonding network (PhOH···O) between tannin and the hydrophilic PEO blocks of F127 (Supplementary Fig. 2). The mechanochemical coordination behaviour between tannin and transition metal ions has been known with the formation of a coordination polymer product (Supplementary Fig. 3)^40^,^41^,^42^. To crosslink tannin around F127 micelles, transition metal acetates were added into the tannin-F127 mixture before ball milling for 30 min, resulting in homogeneous gel-like composites. The tannin-F127-metal composites were finally thermally treated in a N_2_ atmosphere (450–800 °C), during which F127 micelles were completely decomposed and the tannin-metal polymer restructured into a carbon framework (Fig. 1). When Zn(OAc)_2_ was used as a linker, the tannin-Zn polymer with –PhO^−^–Zn^2+^–OPh^−^– coordination becomes a ZnO–carbon hybrid at a moderate temperature such as 450 °C, as observed in the diffraction lines of ZnO in the XRD measurement (Supplementary Fig. 4)^43^. The doped ZnO can be reduced to metallic Zn during further carbonization and then evaporate resulting in pure carbon materials. The evaporation of Zn during calcination at 800 °C was suggested by both inductively coupled plasma-atomic emission spectroscopy (Zn: 0.14 wt%) and XRD results (Supplementary Fig. 5). To optimize the assembly process, a series of mesoporous carbons were prepared from tannin (1 g), surfactants (F127, F88, F87, F68, F38, P123, P103, P85, P65, Triton X-100 and Brij-78) and Zn(OAc)_2_ (3 mmol) composites under various carbonization temperatures. Those carbon materials are denoted as OMC@F127_1_-450, OMC@F127_0.8_-800 and OMC@P123_0.8_-800 and so on, where the subscript word such as ‘F127_0.8_’ corresponds 0.8 g F127 used, while the last number ‘800’ indicates the pyrolysis temperature.

**Figure 2:**
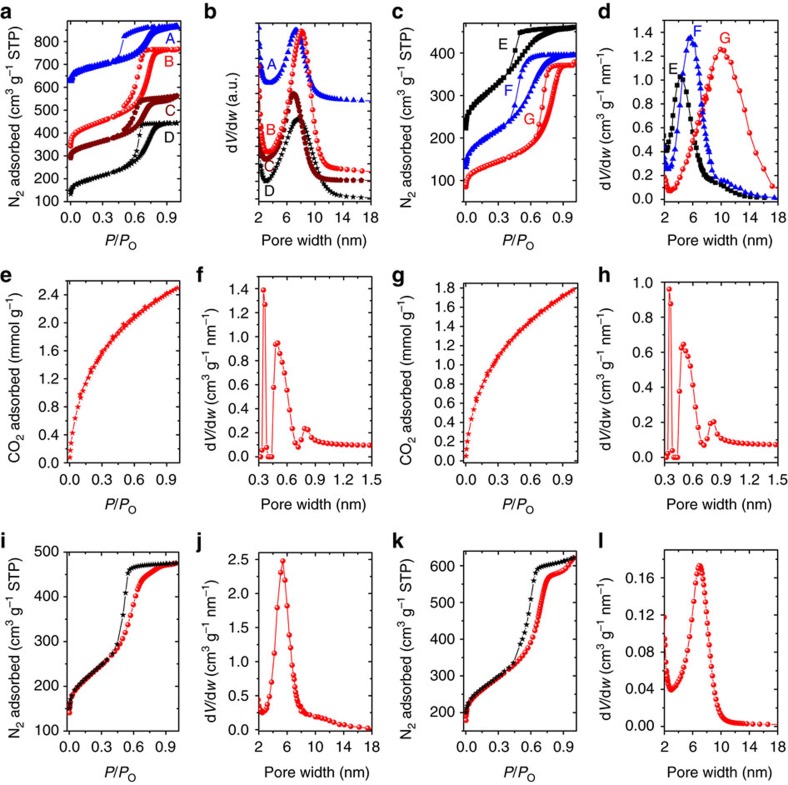
Characterizations of pore structure by gas sorption. (**a**–**d**) N_2_ adsorption–desorption isotherms (77 K) and the corresponding pore size distributions of as-synthesized carbons. A: OMC@F127_0.4_-800, B: OMC@F127_0.6_-800, C: OMC@F127_0.8_-800, D: OMC@F127_1_-800, E: OMC@P65_0.8_-800, F: OMC@F88_0.8_-800 and G: OMC@(F127+Ph_3_P)_0.8_-800. For clarity, the isotherms of A, B, C and E are offset along they axis by 400 cm^3^ g^−1^, 100 cm^3^ g^−1^, 150 cm^3^ g^−1^ and 70 cm^3^ g^−1^, respectively. (**e**,**f**) CO_2_ adsorption–desorption isotherms (273 K) and the corresponding pore size distribution of OMC@F127_0.6_-800 sample. (**g**,**h**) CO_2_ adsorption–desorption isotherms (273 K) and the corresponding pore size distribution of OMC@P123_0.8_-800 sample. (**i**,**j**) N_2_ adsorption–desorption isotherm (77 K) and the corresponding pore size distribution of OMC@P123_0.8_-800 sample. (**k**,**l**) N_2_ adsorption–desorption isotherm (77 K) and the corresponding pore size distribution of Ni-OMC@F127_0.8_-450 sample.

Both F127 and metal salts are necessary for the successful assembly of the mesoporous structure, as suggested by N_2_ adsorption–desorption measurements of the control products at 77 K. The pyrolysis (800 °C) of tannin-F127 (1 g:1 g) or tannin-Zn(OAc)_2_ (1 g:3 mmol) composites derived from ball milling leads to disordered carbons with low pore volumes (0.36 cm^3^ g^−1^ and 0.23 cm^3^ g^−1^, respectively) (Supplementary Fig. 6 and Table 1). Similar disordered carbons with a broad pore size distribution were prepared from the carbonization of tannin acid-cobalt or -iron crystalline complexes^44^. In sharp contrast, carbon OMC@F127_1_-800 derived from tannin-F127-Zn(OAc)_2_ (1 g:1 g:3 mmol) shows an adsorption–desorption pattern of mesoporous structure (pore size: ∼7.8 nm) with an enhanced pore volume (0.69 cm^3^ g^−1^) (Fig. 2a,b, curve D and Supplementary Fig. 6). This result is consistent with the proposed assembly model in which F127 micelles self-assemble to form an ordered mesophase and then Zn(OAc)_2_ crosslinks the tannin units around them.

In addition, the weight ratios of tannin to F127 (1:1, 1:0.8, 1:0.6 and 1:0.4) are flexible. The N_2_ adsorption–desorption measurements of carbon samples (OMC@F127_1_-800, OMC@F127_0.8_-800, OMC@F127_0.6_-800 and OMC@F127_0.4_-800) resulted in type IV isotherms with clear H1 hysteresis loops, a characteristic of mesoporous materials (Fig. 2a, curves A–D). Delayed closure of the hysteresis loop for the OMC@F127_0.4_-800 sample indicates pore constrictions along the main mesopores. Furthermore, the Brunauer–Emmett–Teller (BET)-specific surface areas calculated within the relative pressure range of 0.02–0.05 are in the range of 621–1,057 m^2^ g^−1^ with single point pore volumes reaching 0.76 cm^3^ g^−1^ (Table 1)^45^. The calculated pore size distributions^46^,^47^ are narrow and centred at 6–8 nm, in agreement with the formation of mesopores with widths controlled by the triblock co-polymer templates of carbon materials (Fig. 2b, curves A–D). Such a mesoporous structure in fact can form at 450 °C, but the OMC@F127_1_-450 sample is in fact the mesoporous ZnO-containing polymer (C: 68.11 wt%, H: 3.11 wt% and O: 17.94 wt%). It is understandable since the F127 co-polymer would completely decompose at a temperature above 400 °C in an inert atmosphere^18^. Carbonization temperature also plays a role in controlling the mesopore size. For instance, for the OMC@F127_1_ series (Supplementary Figs 7 and 8 and Table 1), the mesopore widths slightly decreased from 8.6 nm for OMC@F127_1_-450, down to 7.8 nm for OMC@F127_1_-800, indicating a slight shrinkage of the framework with increasing temperature. The amount of Zn(OAc)_2_ (1.8, 2.4, 3.0 and 3.6 mmol) for coordination crosslinking can vary with all loadings resulting in the successful synthesis of mesoporous carbons (Supplementary Fig. 9). To better understand the microporous and ultramicroporous structure of the OMCs, CO_2_ sorption measurements were performed (Fig. 2e–h and Table 1)^48^,^49^,^50^,^51^. For two selected samples, the non local density functional theory (NLDFT)-calculated pore size distributions (PSDs) have maxima for micropore widths at 0.34–0.35 nm, with additional pores of ∼0.50 nm confirming the presence of an important fraction of ultramicroporosity (pores narrower than 0.7 nm)^52^,^53^,^54^. While the origin of these micropores is attributed to the decomposition of thermally unstable organic domains, and evolution of gaseous decomposition products within the carbon walls during carbonization, post-synthesis activation methods are necessary to further increase the micropore content of the present OMC materials^55^.

To adjust the pore size of the carbon products, PEO–PPO–PEO templates with different compositions (Pluronic F88, F87, F68, F38, P123, P103, P85, P65) and nonionic surfactants (PEO-based Triton X-100 and Brij-78) were also explored in the mechanochemical assembly with the tannin-Zn precursor. The assembly with other F-series co-polymers led to mesoporous carbons with pore sizes around 5 nm, slightly smaller than the OMC obtained with F127 (∼7 nm) (Fig. 2c,d, curve F and Supplementary Fig. 10). The pore size roughly increases with the molecular weight of the PPO chain in the F-series co-polymers, which is reasonable since the size of the micelle core will increase with a longer hydrophobic PPO chain (Supplementary Table 1). The P-series co-polymers with a lower weight percentage of PEO are also interesting for the current methodology (Supplementary Fig. 11). For example, the OMC@P65_0.8_-800 sample is composed of small mesopores of ∼4.2 nm (Fig. 2c,d, curve E), while the use of P123 in the self-assembly process can result in carbon with an extremely narrow pore size distribution around 5.4 nm (Fig. 2i,j). In addition to triblock co-polymers, PEO-based nonionic surfactants such as Triton X-100 and Brij-78 were also investigated. The OMC@TritonX-100_0.8_-800 sample is rich in mesopores of ∼5.0 nm (Supplementary Fig. 12). Interestingly, it is also possible to engineer the large mesopores through the use of Brij-78, though the pore size distribution is somewhat broadened (Supplementary Fig. 13). Previously, surfactants other than F127 and P123 had been used as soft templates with limited success and for very restricted conditions^1^,^25^. In addition, the increase of pore size (from ∼6.9 to ∼10.4 nm) could be achieved by adding 10 wt% hydrophobic triphenylphosphine into the F127 for a larger micelle core (Fig. 2c,d, curve G). Because of the abundant library of surfactants available, the mechanochemical self-assembly method can serve as a general method to synthesize mesoporous carbons with tunable pore sizes ranging from 4 to 10 nm.

**Figure 3:**
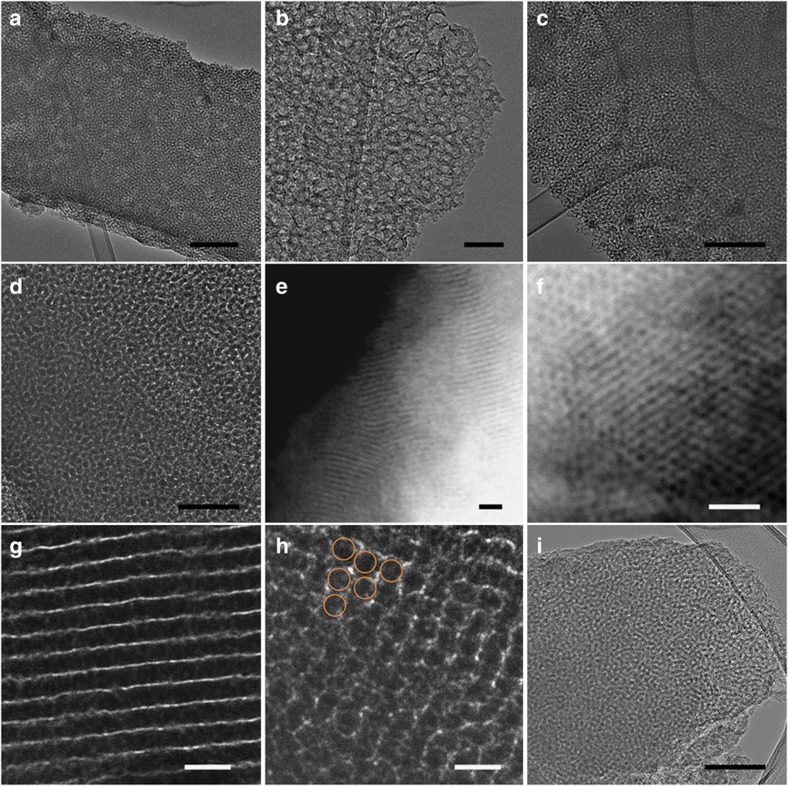
Morphology and structural characterization of OMCs. (**a**,**b**) TEM images of OMC@F127_0.4_-800 sample. Scale bar, 200 (**a**) and 50 nm (**b**). (**c**,**d**) TEM images of OMC@F127_0.6_-800 sample. Scale bar, 200 (**c**) and 100 nm (**d**). (**e**–**h**) STEM-HAADF and TEM images of OMC@F127_0.8_-800 sample. Scale bar, 50 nm (**e**,**f**) and 20 nm (**g**,**h**). (**i**) TEM image of OMC@P123_0.8_-800 sample; scale bar, 100 nm. The orange circles in **h** show the hexagonal array of mesopores.

To further characterize the mesoporous morphology of the as-synthesized carbons, transmission electron microscopy (TEM) and scanning TEM in high angle angular dark field (STEM-HAADF) images were then utilized. As expected, all F127-based carbons show ordered structures yet with different mesophases, which could be tuned by varying the ratio of F127 to the tannin precursor. The OMC@F127_0.4_-800 and OMC@F127_0.6_-800 samples with less F127 yield wormhole architectures and the apparent mesopores of ∼8 nm remain side by side throughout the carbon backbone (Fig. 3a–d and Supplementary Figs 14 and 15). Increasing the mass ratio of F127 in the system resulted in the OMC@F127_0.8_-800 sample with uniform cylindrical mesopores (Fig. 3e–h). The uniformity of the mesopores extends over hundreds of nanometres, as seen in the lower-magnification STEM-HAADF image (Fig. 3e). The long-range 2D hexagonal arrays of mesoporous channels are clear in Fig. 3f,h. The higher magnification image showing the length of the pores (Fig. 3g) illustrates that the pore diameter is in a narrow range of 7.7–8.6 nm, in good agreement with the maximum pore width calculated by N_2_ sorption measurements (Fig. 3g). It is interesting that the carbon wall is quite thin (∼2 nm), which is similar to large pore OMCs obtained from the solution-based soft-templating method. The small angle X-ray scattering (SAXS) data for OMC@F127_0.8_-800 are in agreement with the periodic pore wall mesostructure (Supplementary Fig. 16). The OMC@F127_1_-800 sample with additional F127 possesses a larger mesopore volume but a less ordered structure (Supplementary Fig. 17). The P123-templated carbon shows a cubic-like mesoporous structure (Fig. 3i and Supplementary Fig. 18), while the porosity of the carbons with the nonionic surfactant templates primarily derives from the interstitial voids of particle aggregates (Supplementary Figs 19 and 20). The OMC@(F127-Ph_3_P)_0.8_-800 sample also retains the ordered mesoporous nanoarchitecture, suggesting the possibility of introducing triphenylphosphine into the F127 micelles to enlarge the pore size (Supplementary Fig. 21). Therefore, the mechanochemical assembly of the tannin-Zn coordination network together with F127 is a versatile solid-state self-assembly method for creating hexagonal OMCs based on renewable carbon precursors.

### Fabrication of nickel OMCs

The coordination chemistry of tannin is in fact extremely rich and not limited to Zn^2+^ ion, which can be reduced to the corresponding metal with a low boiling point. It would be ideal if a metal NP-OMC could be directly prepared by introducing transition metals with a high boiling point into the tannin-metal coordination^40^,^55^. Consequently, Ni(OAc)_2_ was then selected for the mechanochemical polymerization process using tannin in the presence of F127. The sample was thermally treated in N_2_ atmosphere to generate hybrid carbon materials (Fig. 1). The carbon materials were denoted as Ni-OMC@F127_0.8_-450, Ni-OMC@F127_0.8_-600 and so on. The appearance of diffraction lines at 2*θ*=44.7°, 52.0° and 76.5° in XRD patterns indicates the presence of metallic Ni in the carbons (Supplementary Fig. 22). We attribute the formation of these metallic Ni NPs to the reduction of NiO by neighbourhood carbons under inert atmosphere^56^, which can take place even at 450 °C. Based on Scherrer’s equation, the average size (5.7 nm) of the Ni crystallites in the Ni-OMC@F127_0.8_-450 is smaller than those in the samples synthesized with both more and less F127 (for example, Ni-OMC@F127_0.6_-450 and Ni-OMC@F127_1_-450) (Supplementary Fig. 22). This observation is likely due to an enhanced confining effect from the hexagonally structured carbon. The N_2_ adsorption–desorption measurement of Ni-OMC@F127_0.8_-450 shows a type IV isotherm with a sharp capillary condensation step at *P*/*P*_0_=∼0.5–0.8 and H1-type hysteresis loop, suggesting the formation of a mesoporous structure (Fig. 2k). Meanwhile, the significant volume adsorbed at low relative pressure (for example, 0–0.1) indicates the presence of abundant micropores inside the carbon wall. The pore size distribution is quite narrow, centring at around 6.9 nm (Fig. 2l). A high specific surface area of 996 m^2^ g^−1^ with a large pore volume of 0.96 cm^3^ g^−1^ is observed, during which the micropores contribute around one half (464 m^2^ g^−1^) (Table 1).

**Figure 4:**
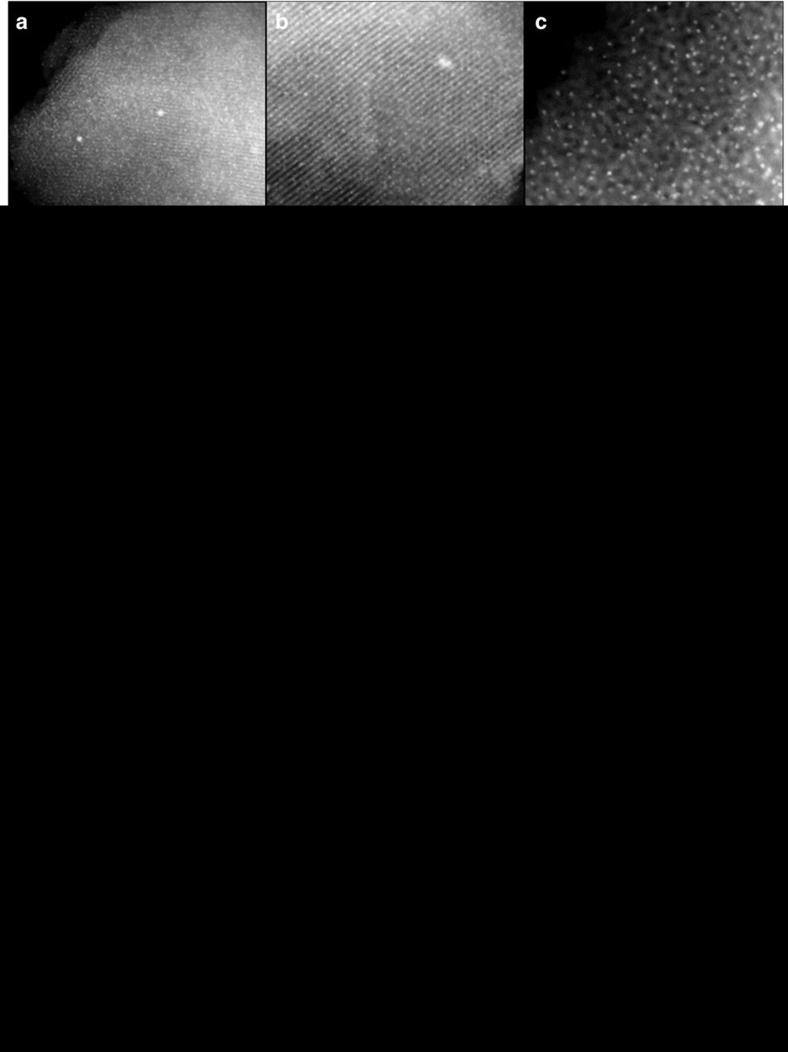
Morphology and structural characterization of nickel OMCs. (**a**–**e**) STEM-HAADF images of Ni-OMC@F127_0.8_-450. Scale bar, 200 (**a**), 100 (**b**), 50 (**c**), 10 (**d**) and 5 nm (**e**). (**f**,**g**) Ni-OMC@F127_0.8_-600. Scale bar, 100 (**f**) and 50 nm (**g**). (**h**) Ni-OMC@F127_0.8_-800. Scale bar, 50 nm. (**i**) Ni-OMC@F127_0.8_-450 recycled from hydrogenation reaction; scale bar, 20 nm. (**j**–**l**) The corresponding particle size distributions. The particle size distribution was calculated based on 150 particles randomly selected. APS, average particle size.

As seen in the STEM-HAADF images, Ni-OMC@F127_0.8_-450 is composed of ordered parallel channels, revealing that Ni^2+^ ions worked well in the mechanochemical assembly with tannin and F127. The apparent pore diameter of ∼7 nm is in excellent agreement with the calculated pore width obtained using the N_2_ sorption measurement (Fig. 4a,b and Supplementary Fig. 23). More importantly, Ni NPs with an average particle size of 4.4 nm (Fig. 4j) are well dispersed throughout the entire OMC domain. (Note that less than 1% Ni NPs with a size of ∼14 nm are observed, which are likely located on the external surface of the OMC). A number of mono-dispersed Ni NPs situated at the end of the cylindrical channels are also observed from the [001] direction (Fig. 4c). High-resolution STEM-HAADF images show that Ni NPs are homogeneously embedded in either the carbon walls or the mesoporous channels (Fig. 4d,e). These Ni NPs, that is, contained within the 1D mesoporous cylinder channel or the carbon wall, should possess good thermal stability because of the constraint of the carbon support which limits aggregation or growth during high-temperature treatment^57^. To explore this confining effect, Ni-OMC samples were also prepared using higher pyrolysis temperatures (600 and 800 °C) (Supplementary Figs 24 and 25). It is worth emphasizing that the Ni NPs within Ni-OMC@F127_0.8_-600 still retain a highly dispersed morphology with an average size of 5.4 nm (Fig. 4f,g,k). A further increase of the temperature (800 °C) promoted the Ni NPs growth to ∼10 nm (Fig. 4h), which may be induced by surface migration and sintering of the Ni NPs at 800 °C. Control samples with Ni on commercial-activated carbon (Ni-AC) or traditional soft-templated OMC (Ni-ST-OMC) were prepared by a wet impregnation method, maintaining the same Ni content as Ni-OMC@F127_0.8_-450 (16.1 wt%). Serious growth and aggregation of the Ni NPs (up to 100 nm) were observed in the Ni-AC and Ni-ST-OMC samples (Supplementary Figs 26–29), and a similar Ni-ST-OMC sample with Ni NPs around 500 nm (15 wt%) was also reported through carbonization of phenolic resin-block co-polymer-nickel nitrate composites^58^. Observations from these control samples indicate that the mechanochemical inorganic–organic assembly process enables NP-OMC composite materials with high NP thermal stability due to excellent confining ability of the OMC support. Current coordination assembly with abundant electron-donating ligands around the Ni^2+^ ions could prevent the migration of Ni NPs formed *in situ* in the carbon matrix, meanwhile those Ni NPs inside the 1D cylinder channels are primarily controlled by space confinement. The exceptional resistance of the Ni-OMCs to sintering during thermal treatment is of great interest in catalysis, especially considering the Ni loading amounts that could be achieved (16.1 wt%).

### Size-sensitive hydrogenation of alkene

The catalytic hydrogenation of alkenes to alkanes is a key process in organic synthesis^59^,^60^. Though many heterogeneous catalysts that promote this selective hydrogenation have been developed, an ideal catalyst, which is active toward large molecules, remains a significant scientific pursuit. One problem with many current catalysts originates from the slow or even difficult mass transfer of large molecules (for example, ∼2 nm) inside narrow pores, preventing the reactant molecules from reaching the active sites smoothly^61^. By coupling the unique properties of OMCs (large pore size, uniform 1D pore channel and high-surface area) with the attractive features of confined Ni NPs (small particle size and high thermal stability and resistance to sintering), the Ni-OMC@F127_0.8_-450 sample synthesized by the mechanochemical approach exhibits most of the prerequisites for an efficient catalyst for the selective hydrogenation of large molecules. To that end, the hydrogenation of cyclohexene, 1-octadecene and cholesteryl acetate, molecules with the same reactive site yet different sizes, was selected as a model reaction to evaluate Ni-OMC@F127_0.8_-450, Ni-AC@450 and Ni-ST-OMC@450 (Table 2). A molecular dynamics simulation in vacuum illustrates their different molecular sizes with the longest distances of 0.50, 1.97 and 1.87 nm (Supplementary Figs 30–32 and Supplementary Note 1). All catalysts exhibited high performance in the transformation of cyclohexene to cyclohexane, suggesting that Ni NPs supported on both microporous or mesoporous supports are accessible to cyclohexene (∼0.5 nm). In the case of 1-octadecene hydrogenation, Ni-OMC@F127_0.8_-450 exhibited a high yield of 1-octadecane while Ni-AC@450 and Ni-ST-OMC@450 gave only moderate yields (65 and 77%). In sharp contrast to the smaller molecules, the hydrogenation of the larger cholesteryl acetate showed different results. The Ni-OMC@F127_0.8_-450 offered a good yield of 92%, but limited yields were observed for the Ni-AC@450 and Ni-ST-OMC@450 catalysts. These results indicate that large molecules, such as 1-octadecene and cholesteryl acetate, can be freely adsorbed on active Ni NPs of Ni-OMC@F127_0.8_-450. However, activated carbon or post-impregnated OMC are composed of either micropores (Supplementary Fig. 33) or mesopores that are blocked by large Ni NPs. In both cases, the bulky cholesteryl acetate with a rigid backbone was likely prevented from entering the pores and accessing the active sites. Therefore, Ni-OMC@F127_0.8_-450 has been proven to be efficient toward the hydrogenation of various molecules (Supplementary Table 2). Moreover, the catalyst can be magnetically recycled and reused at least five times without any loss of activity (Supplementary Table 3). Further, the Ni NPs in the recycled catalyst showed only a slight increase in size from 4.35 to 6.24 nm after recycling (see the STEM-HAADF images in Fig. 4i,l), once again revealing the excellent stability of Ni-OMC@F127_0.8_-450.

## Discussion

In summary, a general, environmentally friendly, high throughout, mechanochemical synthesis of OMC and Ni-OMC has been realized, based on two fortuitous findings: (1) The coordination polymerization between polyphenols and metal ions can provide a versatile alternative to traditional phenol-formaldehyde polycondensation, and this process can replace the toxic organic precursors (for example, phenol, resorcinol or phloroglucinol) with biomass-derived tannin and eliminate the use of carcinogenic crosslinkers, that is, formaldehyde. Other transition metal ions with vacant orbital that readily coordinate to catechol are potential metal species for the current assembly. Various metal NPs/oxides/carbides could in principle be *in situ* confined inside the OMCs, adding another dimension to enrich the current OMC library. (2) The mechanochemical synthesis that runs under solid conditions can guarantee the crosslinking polymerization undergoing within the voids of the F127 mesophases, shortening the assembly time to 1 h, much less than the processing time (1–3 days) of the solution route^62^. The solid-state assembly also avoids solvent wastes (for example, water, ethanol or tetrahydrofuran), and favourably allows the use of precursors with no or low solubility in solvents.

OMCs with hexagonal cylindrical structures have been prepared with large pore size (∼8 nm), narrow pore size distribution and high pore volume (up to 0.96 cm^3^ g^−1^). Importantly, the coordination polymer allows *in situ* reduction of Ni^2+^ ions into Ni NPs, which are also confined and highly dispersed in the OMCs. Those Ni-OMCs show excellent thermal stability up to 600 °C (Ni NPs: ∼5.4 nm). Ni-OMCs were also successfully used for the selective hydrogenation of large molecules (∼2 nm) with high yield, once again highlighting the unique features of the mesoporous catalysts. Meanwhile, the introduction of mechanochemical synthesis by ball milling together with the inexpensive, abundant and biomass-derived tannin precursor (<$20 per kg) make the OMC-based technologies promising for practical applications (Supplementary Table 4). In addition to tannin, tannin derivatives, such as ellagotannins, gallotannins, quercetin and ellagic acid, and low-weight molecule lignin are also potential resources for OMC production.

## Methods

### General methods and characterization

N_2_ adsorption–desorption analysis was performed at 77 K using a TriStar (Micromeritics Instrument Corp.; Norcross, GA, USA), equipped with automated surface area and pore size analyser. Before analysis, samples were degassed at 200 °C for 24 h. CO_2_ adsorption isotherms were acquired at 273 K using a Quantachrome Autosorb-1. Before analysis, samples were allowed to outgas at 200 °C under turbomolecular vacuum pumping for a minimum of 15 h. For TEM and STEM characterization, samples were dispersed in ethanol using an ultrasonic bath. The final suspensions were transferred to TEM grids and dried in ambient air before electron microscopy analysis. The material morphology was visualized using STEM-HAADF images and electron energy loss spectroscopy on a Nion UltraSTEM 200 microscope operated at 200 kV. Powder X-ray diffraction (XRD) and SAXS were measured on a Bruker D8 diffractometer equipped with scintillation counter. The Zn and Ni contents were measured by inductively coupled plasma-atomic emission spectroscopy. All gas chromatography experiments were carried out and recorded by Agilent Technology 7890A. Fourier transform infrared spectrum was collected by PerkinElmer Frontier Fourier transform infrared spectrometer. Mimosa tannin from SILVACHIMICA S.r.l. (Italy) was used as the carbon precursor.

### Synthesis of OMC and Ni-OMC by ball milling

In a typical synthesis, tannin (1 g) and F127 (0.4–1 g) were added to a commercially available 4.5 cm (diameter) by 5.5 cm (height) screw-capped stainless steel reactor along with six stainless steel ball bearings (2 × diameter 1.2 cm; 2 × diameter 0.7 cm; 2 × diameter 0.5 cm. total weight: 230 g). The reactor was placed in a high-speed vibrating ball miller (1,200 rounds min^−1^, 300 W motor power) and the mixtures were ball milled for 30 min. Then, Zn(OAc)_2_·H_2_O or Ni(OAc)_2_·H_2_O (3 mmol) was added into the reactor for ball milling in another 30 min. The resulting gel-like products were washed with deionized H_2_O and ethanol, followed by drying at room temperature overnight. The composites were finally carbonized at 450 °C (heating rate: 5 K min^−1^, holding time: 2 h), or at 600/800 °C (5 K min^−1^ to 450 °C for 2 h and then 5 K min^−1^ to final temperature for a holding time of 2 h) under an inert N_2_ atmosphere (N_2_ 200 ml min^−1^). The synthesis with other surfactants follows the same process.

### Selective hydrogenation of different sized molecules

The selective hydrogenation was carried out in a stainless steel batch reactor (Parr Instrument, USA). Typically, cyclohexene or 1-octadecene (1 mmol), decane (internal standard, 1 mmol), ethanol (3 ml) and Ni catalyst (10 mg) were loaded into the reactor (total volume: 100 ml). The reactor was sealed and purged with H_2_ to replace the air for four times. The H_2_ pressure was kept at 3 MPa, and the reactor was heated to 130 °C for 10 min. Then, the hydrogenation reaction was carried out for 2 h with magnetic stirring (stirring rate: 800 r.p.m.). After reaction, the reactor was placed in cold water to quench the reaction. The oxidation products were analysed by gas chromatography. Due to the solubility issue, hydrogenation of cholesteryl acetate was carried out in acetone solvent. Cholesteryl acetate (0.5 mmol, 0.214 g), acetone (10 ml) and Ni catalyst (10 mg) were loaded into the reactor (total volume: 100 ml), followed by a same process as above. After completion of the reaction, the solvent was removed under reduced pressure at 45 °C. The residue was purified by silica gel column chromatography (ethyl acetate/petroleum ether=1/11) to afford 3β-acetoxy-5α-cholestane as white powder. The product was analysed by ^1^H-NMR spectroscopy in CDCl_3_ and elemental analysis. ^1^H-NMR (500 MHz, CDCl_3_): *δ*=0.64 (s, 3H, 18-CH_3_), 0.85 (d, *J*=6.6 Hz, 6H, 26-CH_3_, 27-CH_3_), 0.90 (d, *J*=6.5 Hz, 3H, 21-CH_3_), 1.20 (s, 3H, 19-CH_3_), 2.03 (s, 3H, 29-CH_3_CO), 4.64 (m, 1H, 3-CH).

### Data availability

All relevant data are available from the authors on reasonable request.

## Additional information

**How to cite this article:** Zhang, P. *et al*. Solid-state synthesis of ordered mesoporous carbon catalysts via a mechanochemical assembly through coordination cross-linking. *Nat. Commun.* **8,** 15020 doi: 10.1038/ncomms15020 (2017).

**Publisher’s note**: Springer Nature remains neutral with regard to jurisdictional claims in published maps and institutional affiliations.

## Supplementary Material

Supplementary Information Supplementary Figures, Supplementary Tables, Supplementary Note and Supplementary References

## Figures and Tables

**Table 1:** Calculated N_2_ at 77 K adsorption parameters for the various tannin-based materials obtained with or without metal crosslinkers, using different triblock co-polymers as templates, and under various carbonization temperatures.

**Sample**	***V***_**SP**_ **(cm**^**3**^** g**^**−1**^)*	***S***_**BET**_ **(m**^**2**^** g**^**−1**^**)**†	***V***_**mi**_ **(cm**^**3**^** g**^**−1**^**)**‡	***S***_**mi**_ **(m**^**2**^** g**^**−1**^**)**§	***w***_**KJS**_ **(nm)**||	***V***_**mi**_ **CO**_**2**_ **(cm**^**3**^** g**^**−1**^**)**¶
C@Tannin-Zn	0.23	514	0.20	469	–	–
C@Tannin-F127	0.36	395	0.11	245	–	–
OMC@F127_0.4_-800	0.59	773	0.19	475	7.3	0.22
OMC@F127_0.6_-800	0.76	1057	0.24	601	7.8	0.29
OMC@F127_0.8_-800	0.58	621	0.12	293	6.9	0.29
OMC@F127_1.0_-450	0.66	547	0.08	180	8.6	–
OMC@F127_1.0_-600	0.67	869	0.17	412	8.2	–
OMC@F127_1.0_-800	0.69	734	0.16	390	7.8	0.18
Ni-OMC@F127_0.8_-450	0.96	996	0.19	464	6.9	0.18
Ni-OMC@F127_0.8_-600	0.73	769	0.15	356	7.8	–
Ni-OMC@F127_0.8_-800	0.52	558	0.14	355	9.2	–
OMC@F38_0.8_-800	0.49	722	0.16	381	5.3	–
OMC@F68_0.8_-800	0.58	770	0.15	350	5.3	–
OMC@F87_0.8_-800	0.62	765	0.17	412	5.9	–
OMC@F88_0.8_-800	0.61	733	0.13	316	5.7	–
OMC@P65_0.8_-800	0.60	851	0.15	340	4.2	–
OMC@P85_0.8_-800	0.66	770	0.17	405	6.6	–
OMC@P103_0.8_-800	0.76	825	0.19	466	7.5	–
OMC@P123_0.8_-800	0.73	811	0.13	310	5.4	–
OMC@Bj78_1_-800	0.89	695	0.16	382	17	–
OMC@TritonX100_0.8_-800	0.50	782	0.17	407	5.0	–
OMC@(F127+Ph_3_P)_0.8_-800	0.57	496	0.10	244	10.4	–

^*^Single point pore volume at relative pressure of 0.98.

^†^Specific surface area calculated using the BET equation in the relative pressure range of 0.02–0.05.

^‡^Micropore volume.

^§^Micropore surface area calculated using the carbon black STSA *t*-plot equation within the thickness range of 0.354–0.500 nm.

^||^Pore width from the distribution maxima calculated according to the KJS method^47^ using carbon black as reference.

^¶^Cumulative plot from NLDFT analysis for CO_2_ isotherms for pores up to 1.5 nm.

**Table 2:**
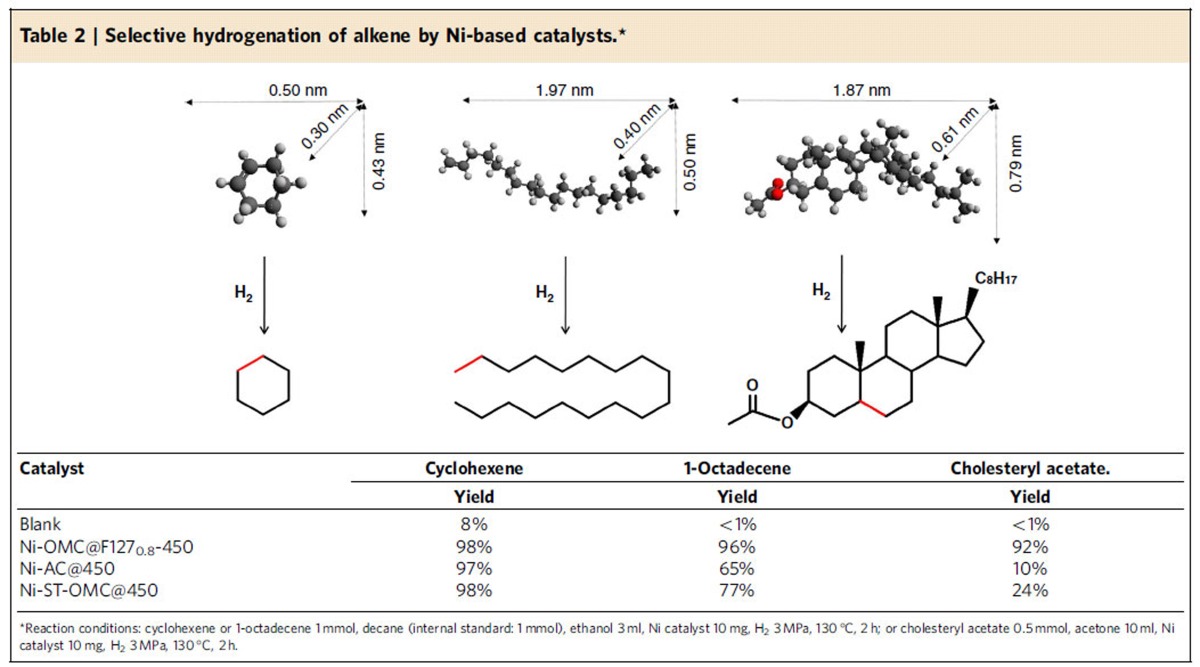
Selective hydrogenation of alkene by Ni-based catalysts.^*^
