# Supplementary material for: Solid-state synthesis of ordered mesoporous carbon catalysts via a mechanochemical assembly through coordination cross-linking
Source: Nat Commun. 2017 Apr 28;8:15020. doi: 10.1038/ncomms15020 (PMC5477501; doi:10.1038/ncomms15020)
Supplement: Supplementary Information — Supplementary Figures, Supplementary Tables, Supplementary Note and Supplementary References [file ncomms15020-s1.pdf]

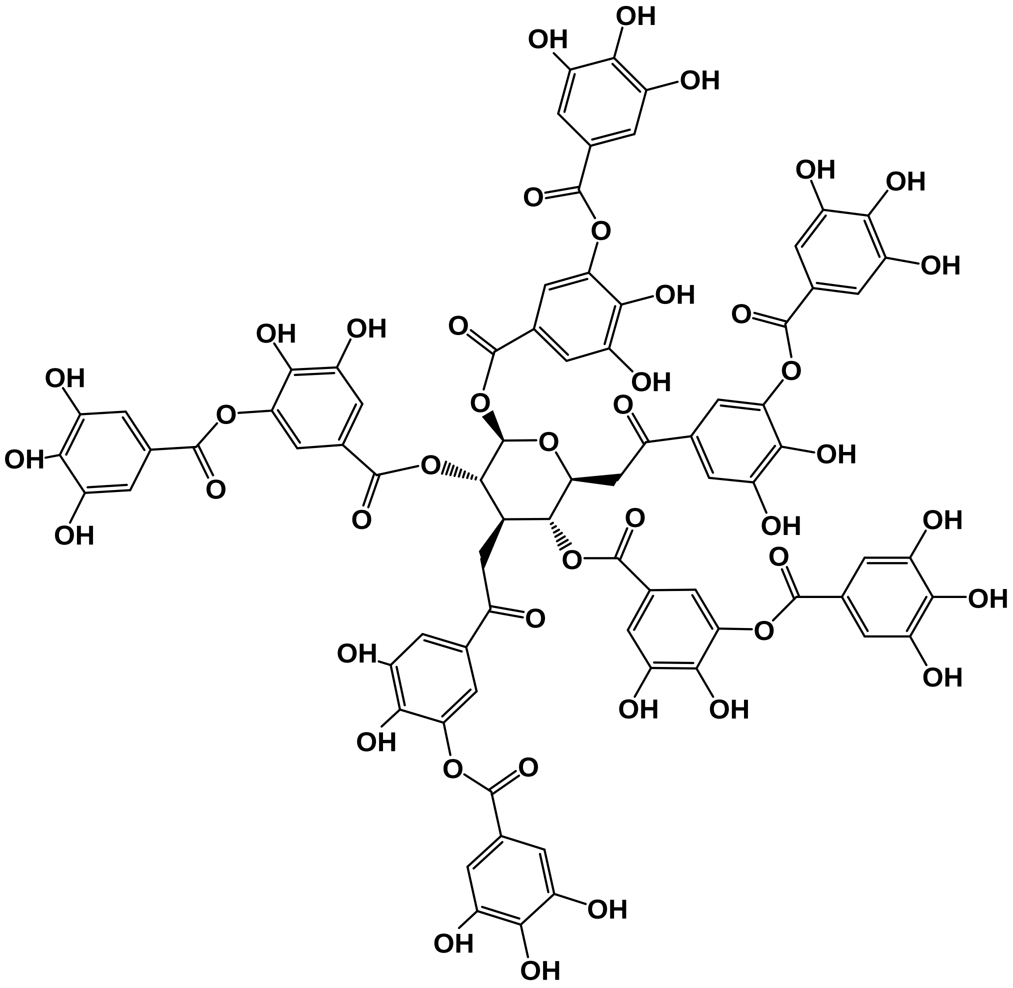

Supplementary Figure 1. The chemical structure of tannin acid (CAS: 1401-55-4)—a typical form of tannins.

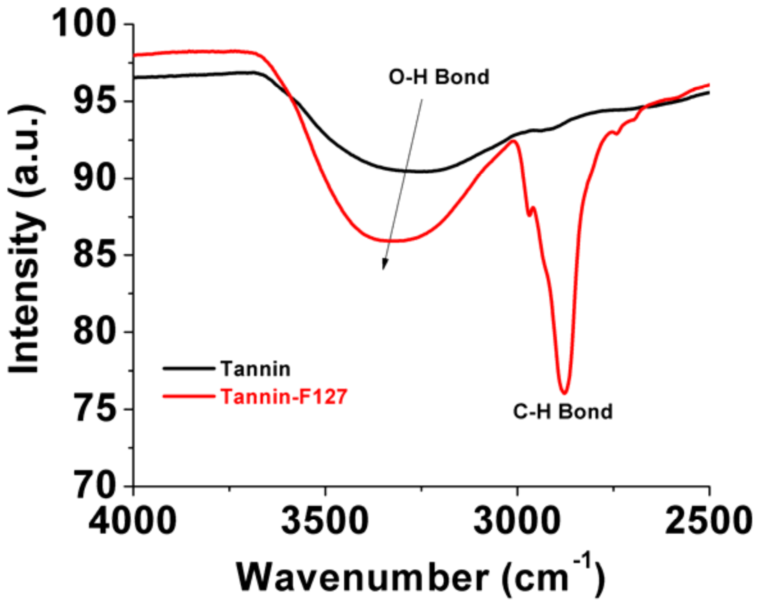

Supplementary Figure 2. FTIR spectra of tannin, and tannin-F127 composite (1 g-1 g) by ball milling for 0.5 h.

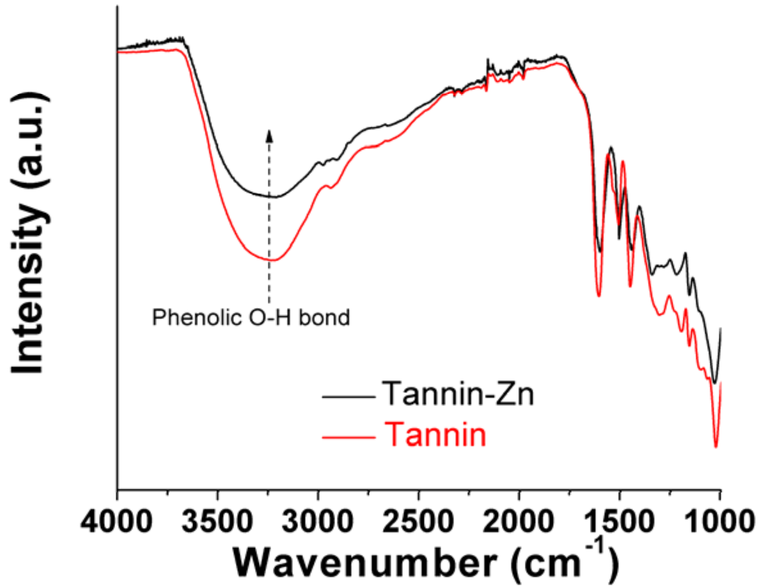

Supplementary Figure 3. Fourier transform infrared (FTIR) spectra of tannin, and tannin-Zn polymer (1 g-3 mmol) by ball milling for 0.5 h. The coordination between tannin and Zn^{2+} ion was suggested by the decrease of phenolic O-H vibrational peaks in FTIR for tannin-Zn sample, though a hump peak related to -OH group remained. There are still one or two -OH groups left in the pyrogallol- or catechol-like rings after the coordination polymerization.

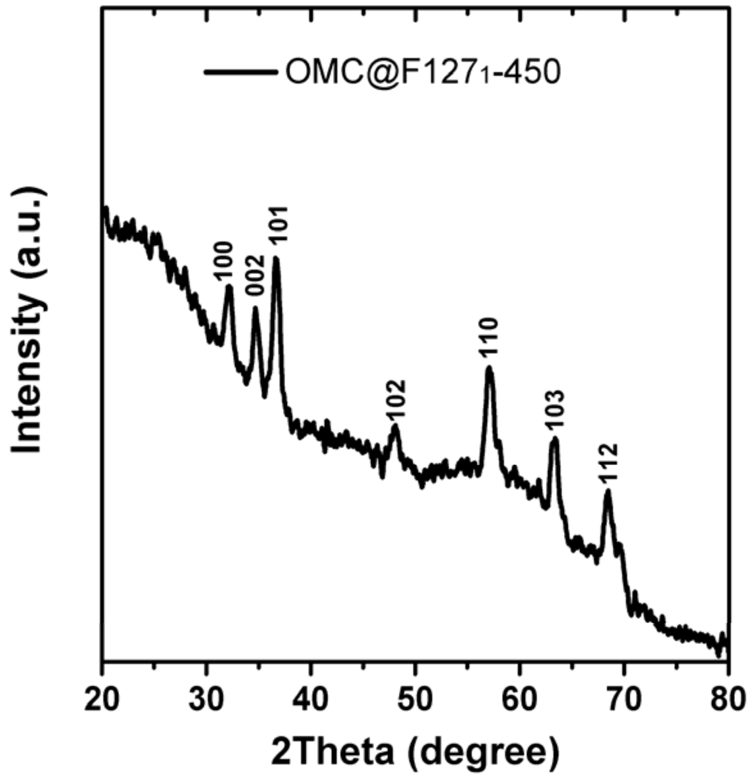

Supplementary Figure 4. XRD pattern of OMC@F127$_1$-450 sample. The diffraction peaks match well with ZnO (JCPDS: 36-1451), revealing that the tannin-Zn polymer with -PhO-Zn-OPh- coordination has transformed into ZnO-carbon at 450°C.

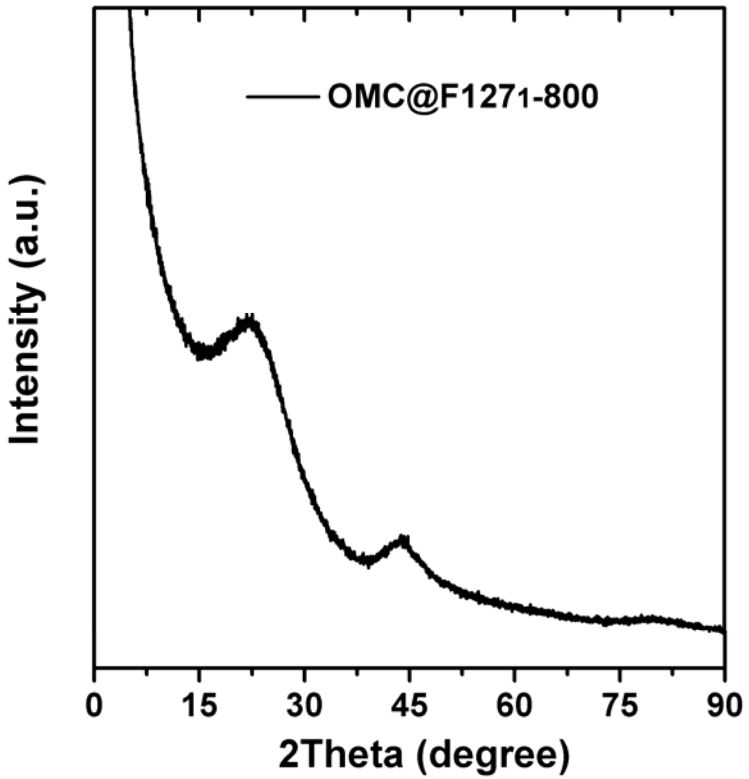

Supplementary Figure 5. XRD pattern of OMC@F127$_1$-800 sample. The broad peaks at ~ 22° and ~44° correspond to the (002) and (100) reflection of the graphitic-type amorphous carbon. No responses relating to Zn or ZnO have been observed, suggesting the evaporation of Zn during high temperature carbonization.

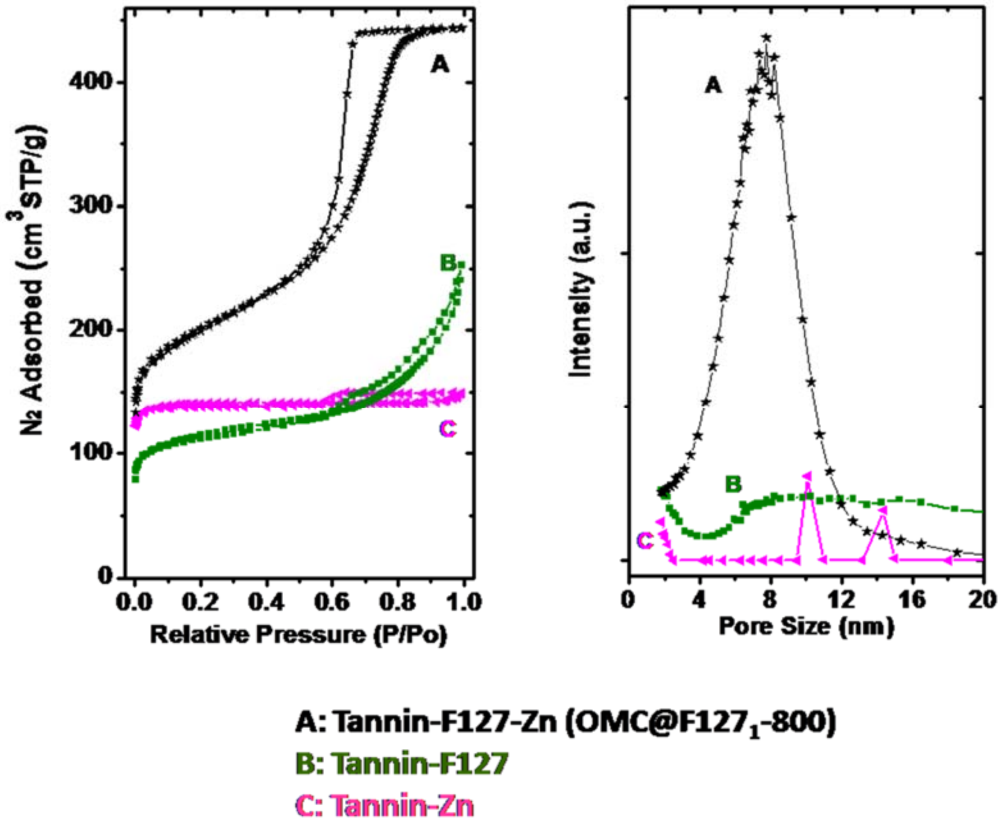

Supplementary Figure 6. Nitrogen adsorption-desorption isotherms (77 K) of control samples and the pore size distributions.

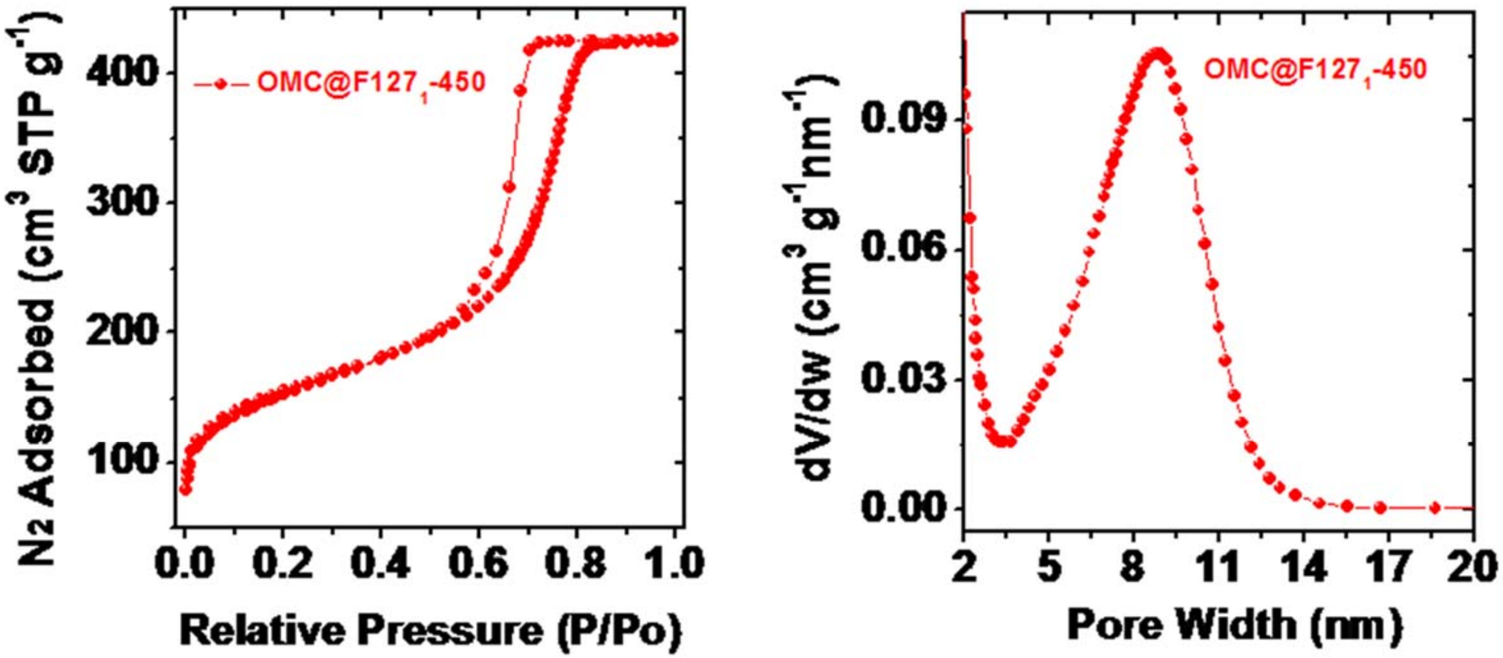

Supplementary Figure 7. Nitrogen adsorption-desorption isotherm of OMC@F127$_1$-450 sample at 77 K. A narrow pore size distribution around 8 nm was got. The ordered mesopores have already formed by calcination at 450°C.

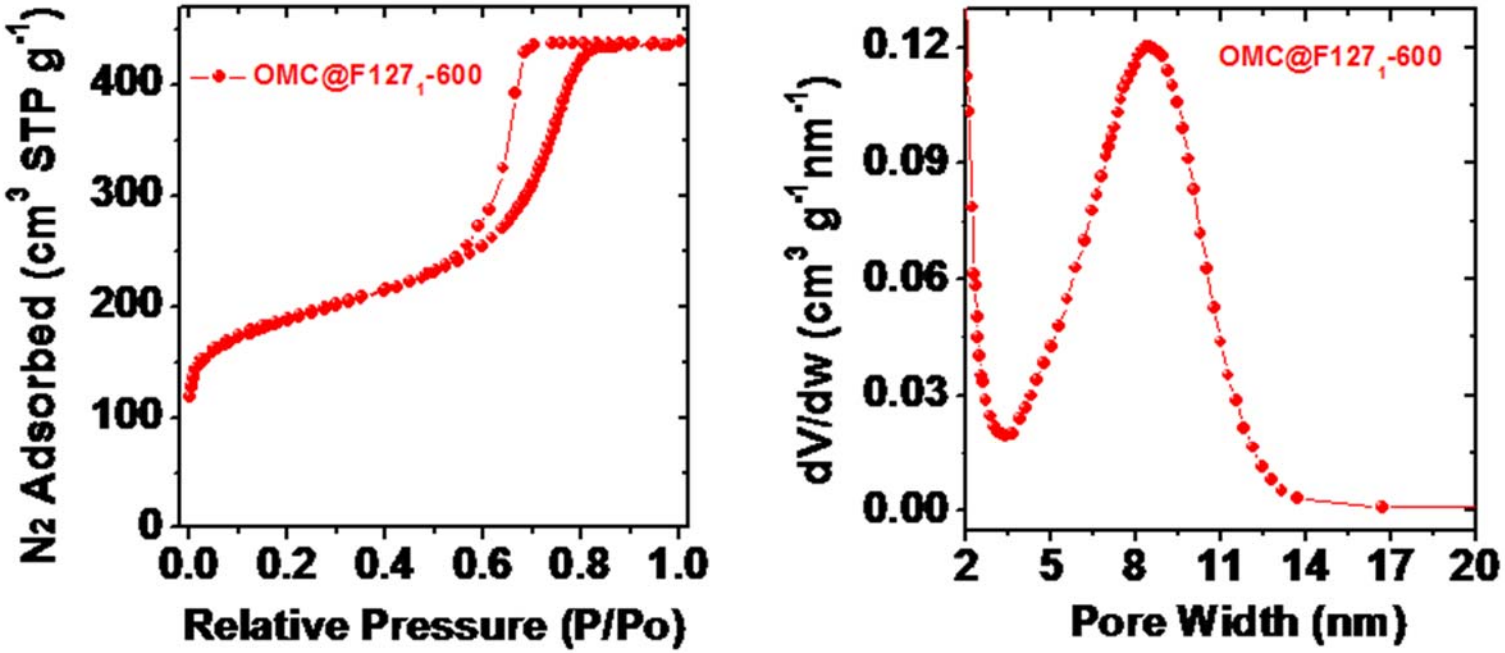

Supplementary Figure 8. Nitrogen adsorption-desorption isotherm of OMC@F127$_1$-600 sample at 77 K. The pore size distribution is quite similar with the curve of OMC@F127$_1$-450 sample. However, there is a clear increase on the N_2 uptake at low relative pressure (P/Po= 0-0.05), induced by the formation of more micropores within carbon walls during polymer decomposing.

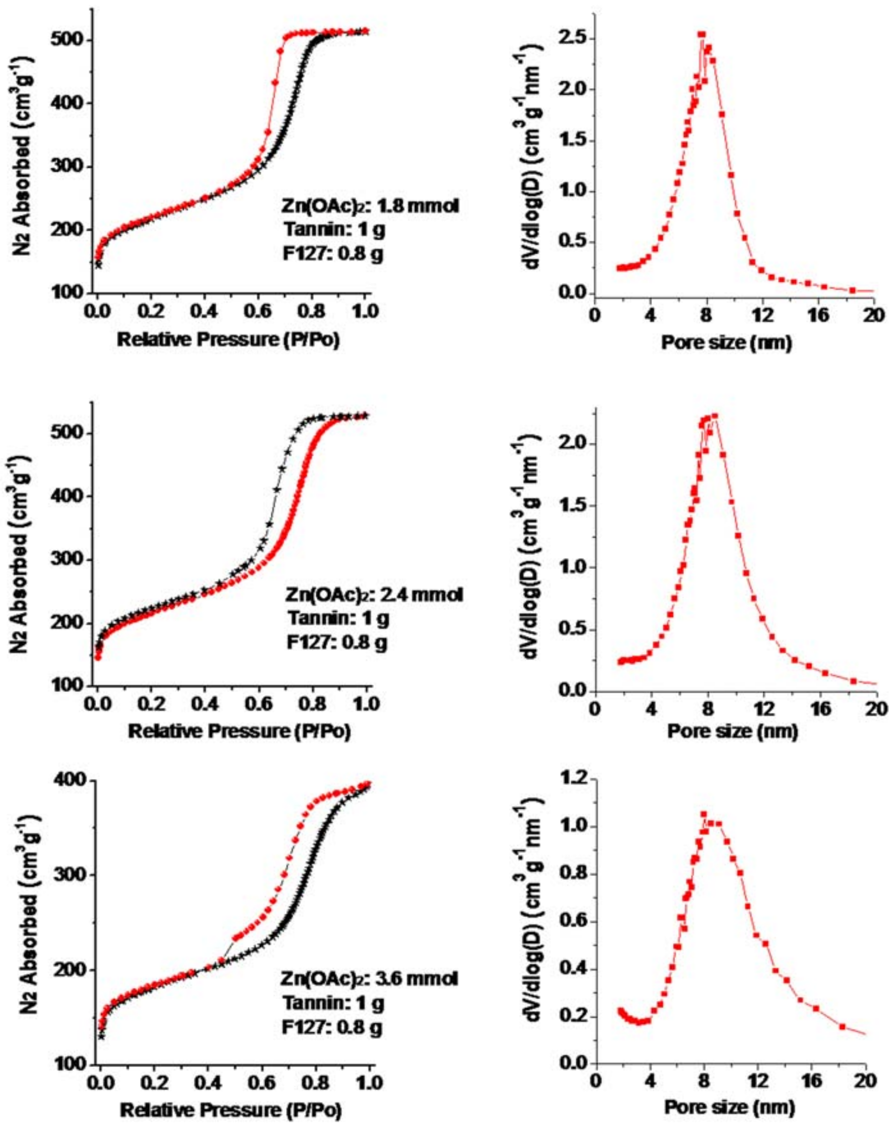

Supplementary Figure 9. Nitrogen adsorption-desorption isotherms of those carbons by different $Zn(OAc)_2$ amounts (calcination at 800°C). $Zn(OAc)_2$ (1.8, 2.4, 3.0 or 3.6 mmol) was added into the tannin-F127 composite (1g-1g) for coordination assembly. The specific surface areas of those carbon samples are 762, 753, 734 and 638 cm^2g^{-1}, respectively. The pore size distributions for $Zn(OAc)_2$ amounts of 1.8, 2.4 and 3.0 mmol are narrower than the one with 3.6 mmol $Zn(OAc)_2$. Here, 3 mmol $Zn(OAc)_2$ is selected as an optimized amount since lower amounts will decrease the carbon yield due to an uncompleted coordination with tannin. The isotherm of the carbon with 3 mmol $Zn(OAc)_2$ is shown in Figure 2a-2b, Curve C.

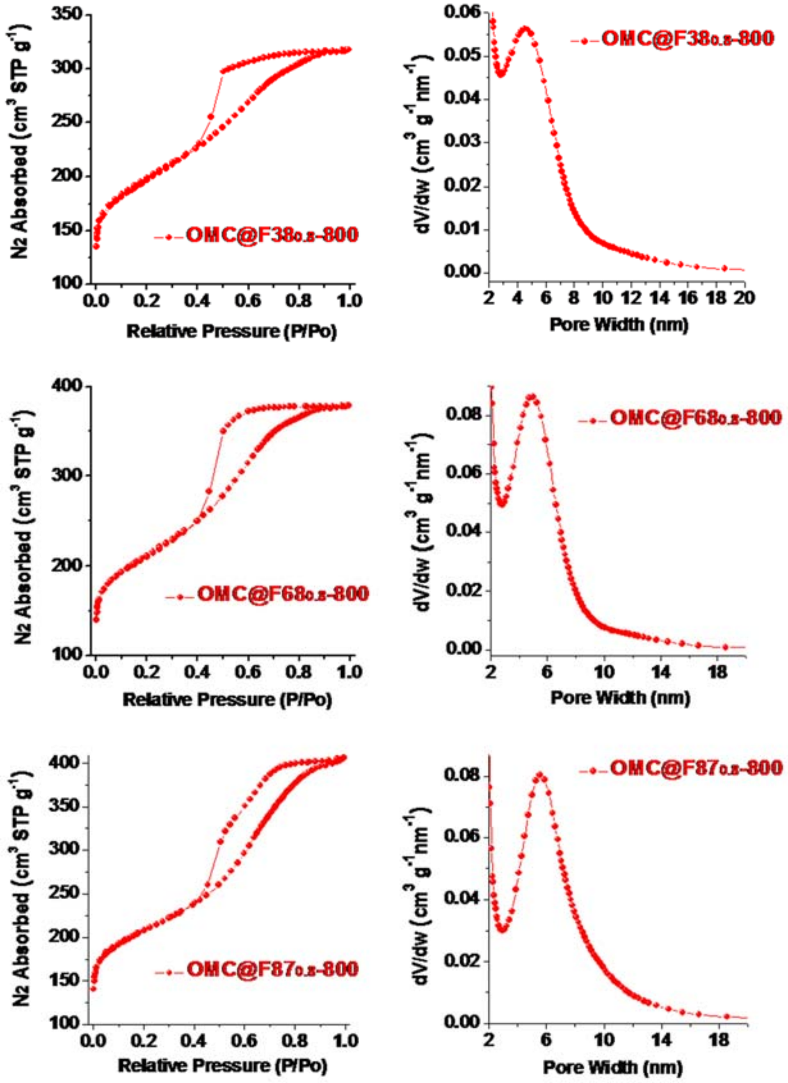

Supplementary Figure 10. Nitrogen adsorption-desorption isotherms and pore size distributions of carbon samples from F-series copolymers.

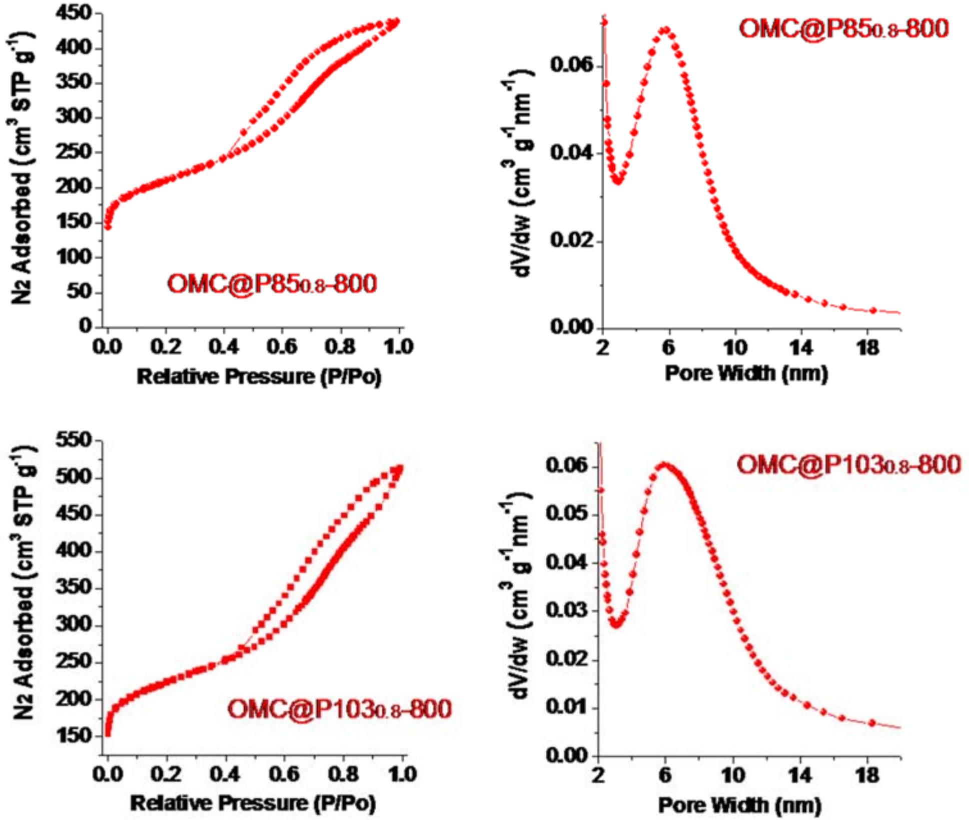

Supplementary Figure 11. Nitrogen adsorption-desorption isotherms (77 K) and pore size distributions of carbon samples templated by P85 and P103.

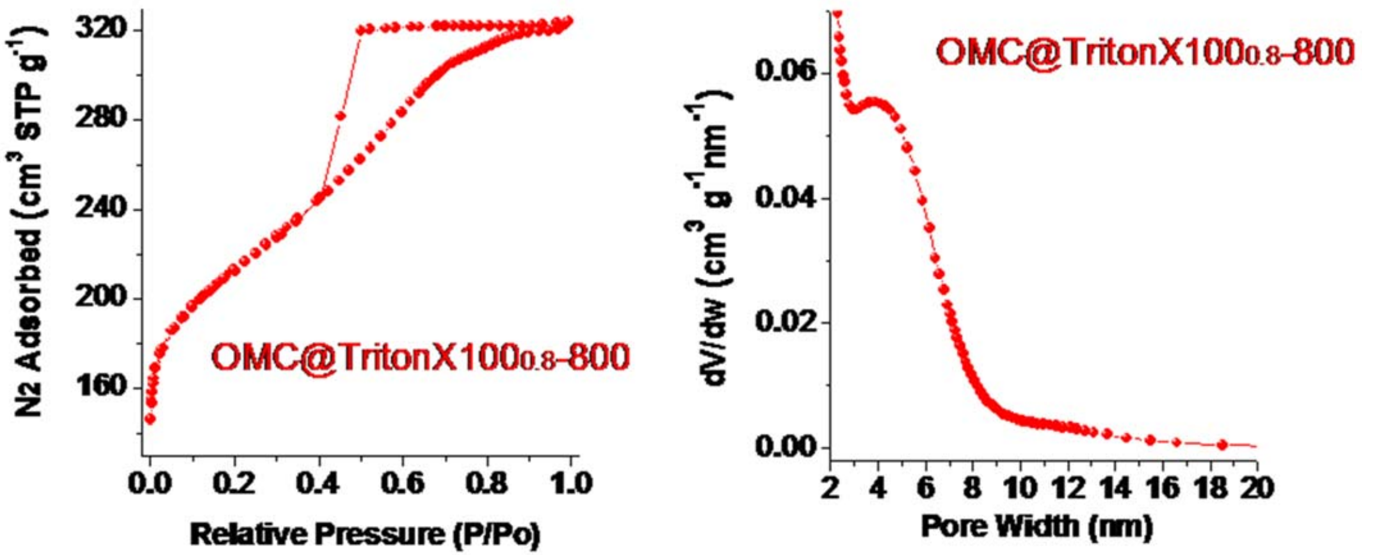

Supplementary Figure 12. The nitrogen adsorption-desorption isotherm (77 K) and pore size distribution of carbon sample by Triton X-100 surfactant. The isotherm curve presents a type IV hysteresis with a condensation step around P/Po= 0.5, while the pore size is centered at ~5 nm.

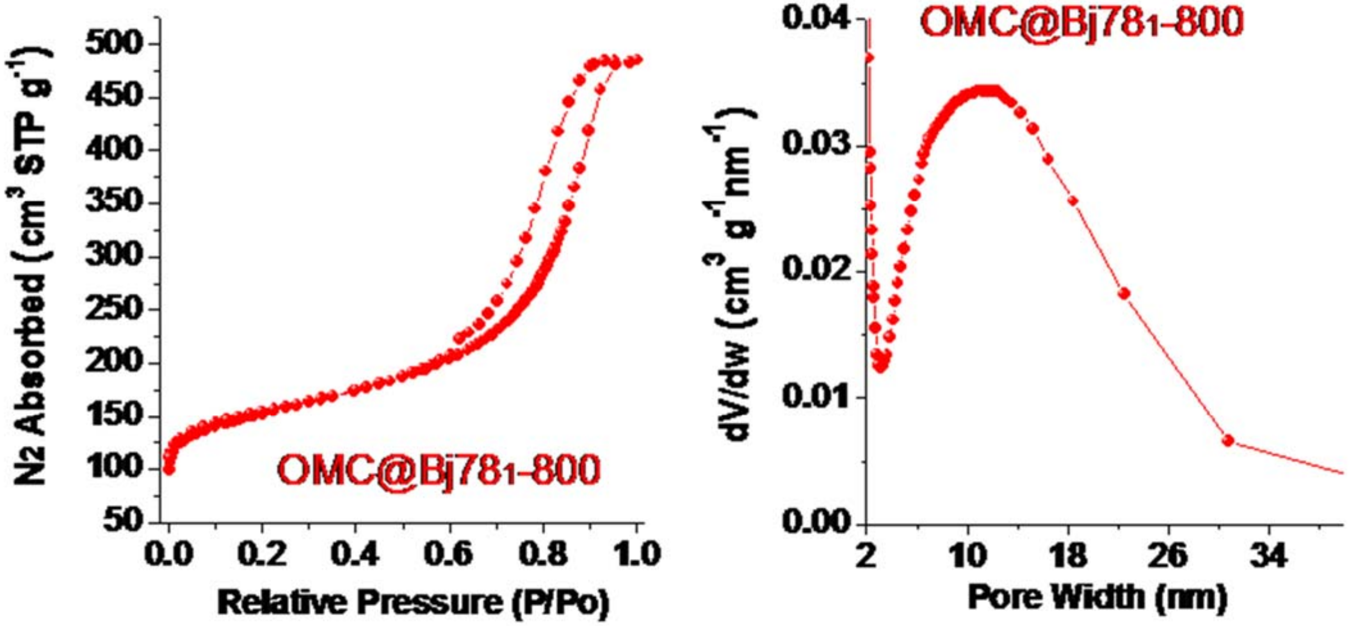

Supplementary Figure 13. The nitrogen adsorption-desorption isotherm (77 K) and pore size distribution of carbon sample with Brij-78 surfactant. The OMC@Bj78$_1$-800 sample shows a very broad pore size from 5 to 30 nm, suggesting the poor ordered textural structure.

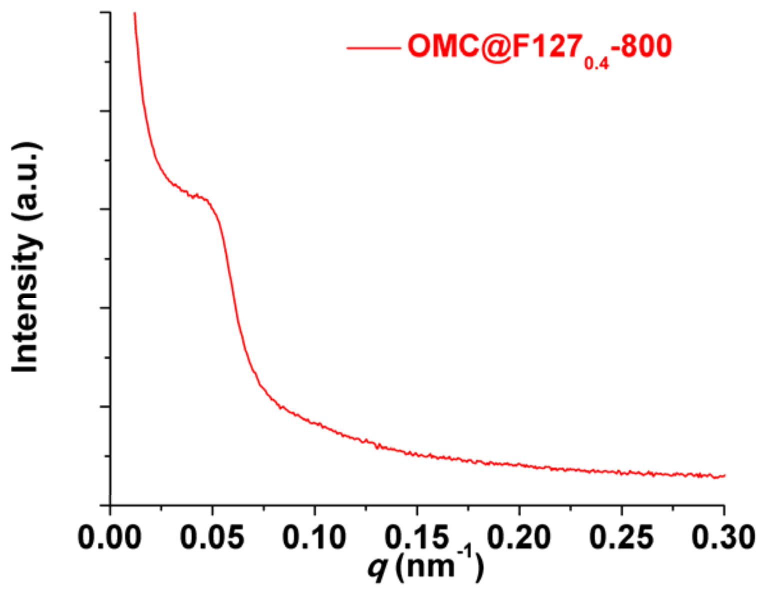

Supplementary Figure 14. SAXS pattern of OMC@F127$_{0.4}$-800 sample.

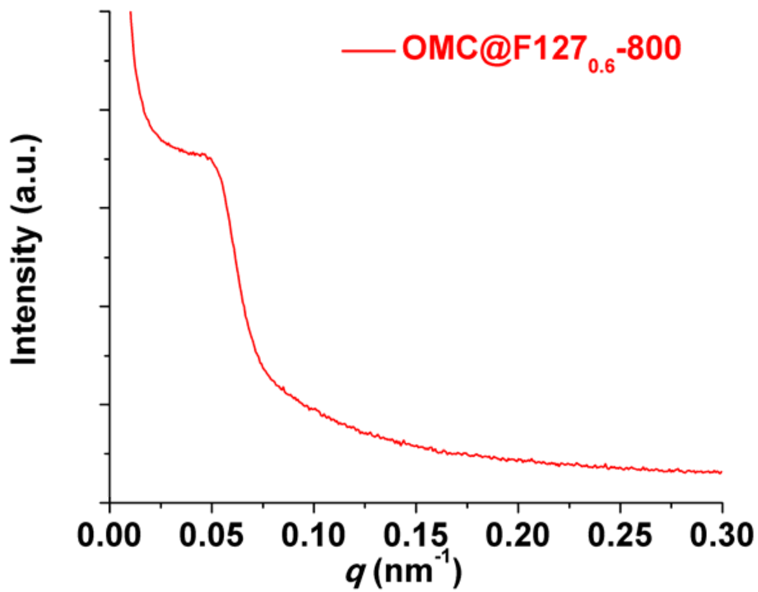

Supplementary Figure 15. SAXS pattern of OMC@F127$_{0.6}$-800 sample.

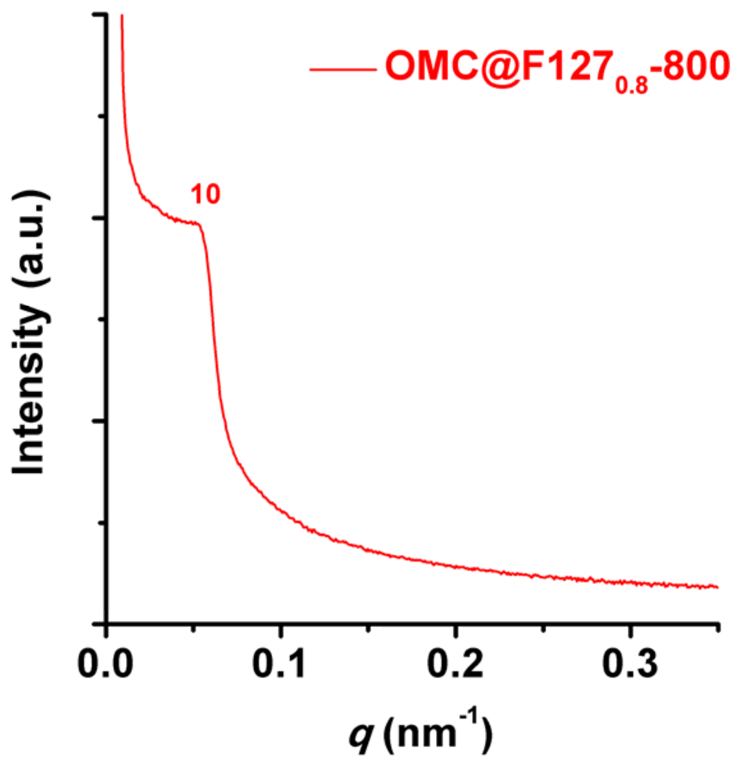

Supplementary Figure 16. SAXS pattern of OMC@F127$_{0.8}$-800 sample.

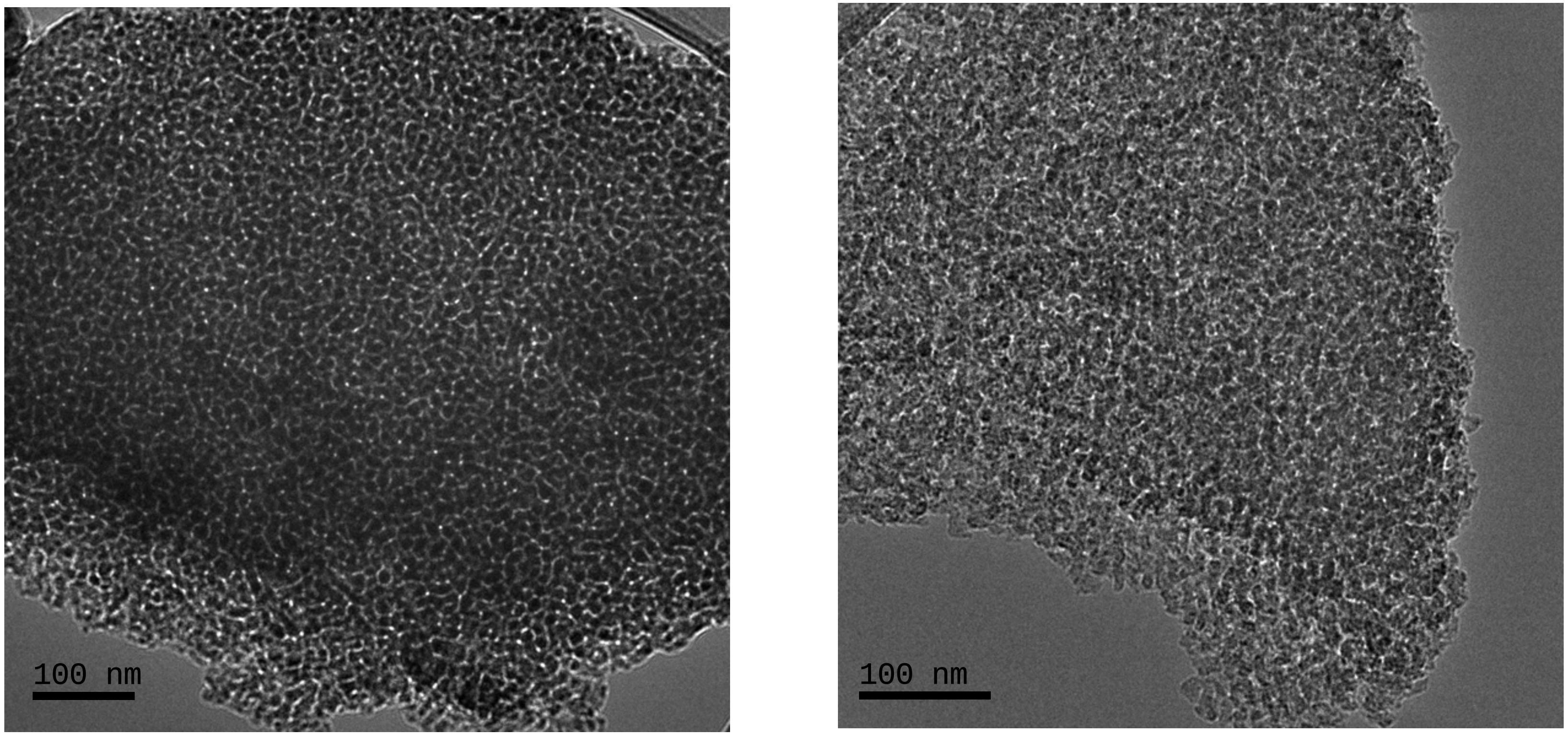

Supplementary Figure 17. TEM images of OMC@F127$_1$-800 sample.

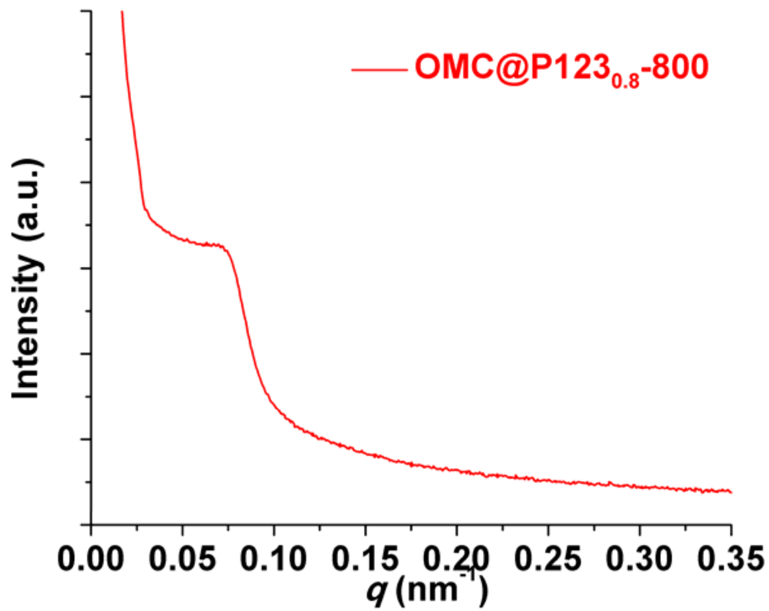

Supplementary Figure 18. SAXS pattern of OMC@P123$_{0.8}$-800 sample.

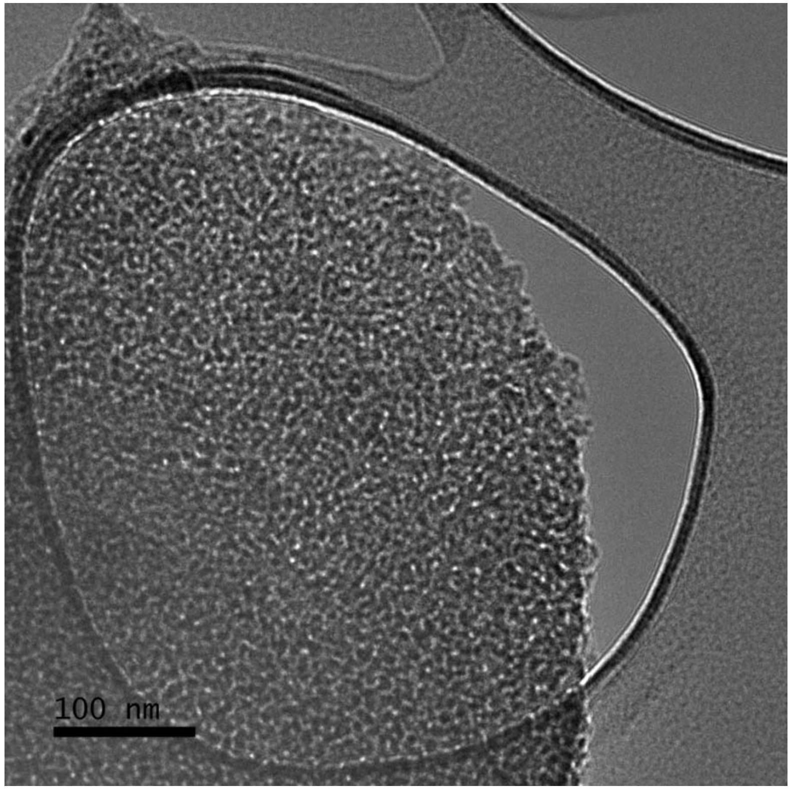

Supplementary Figure 19. A typical TEM image of OMC@TritonX-$100_{0.8}$-800 sample.

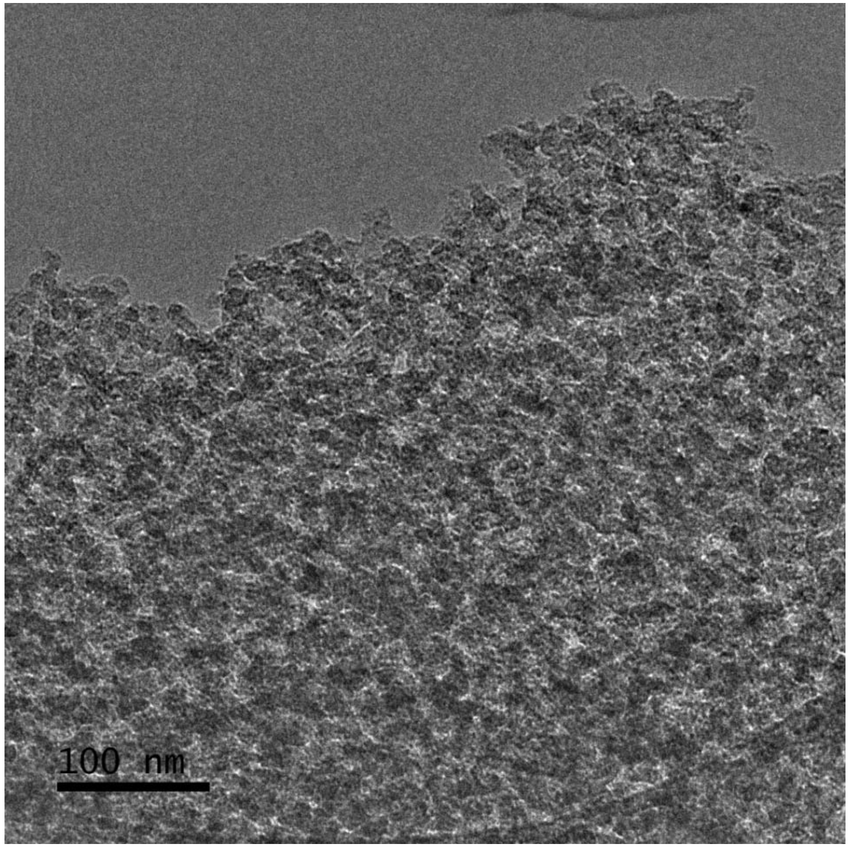

Supplementary Figure 20. A typical TEM image of OMC@Bj78$_1$-800 sample.

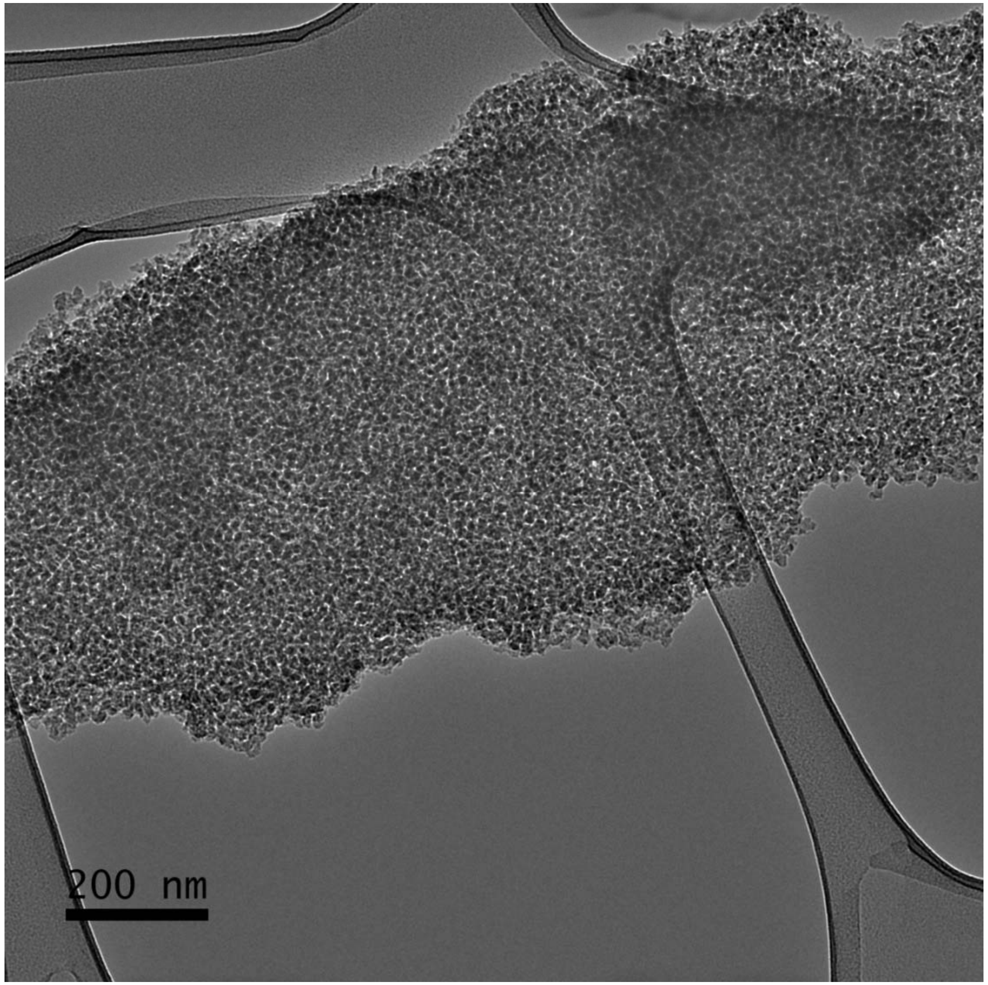

Supplementary Figure 21. A typical TEM image of OMC@(F127-Ph_3P)$_{0.8}$-800.

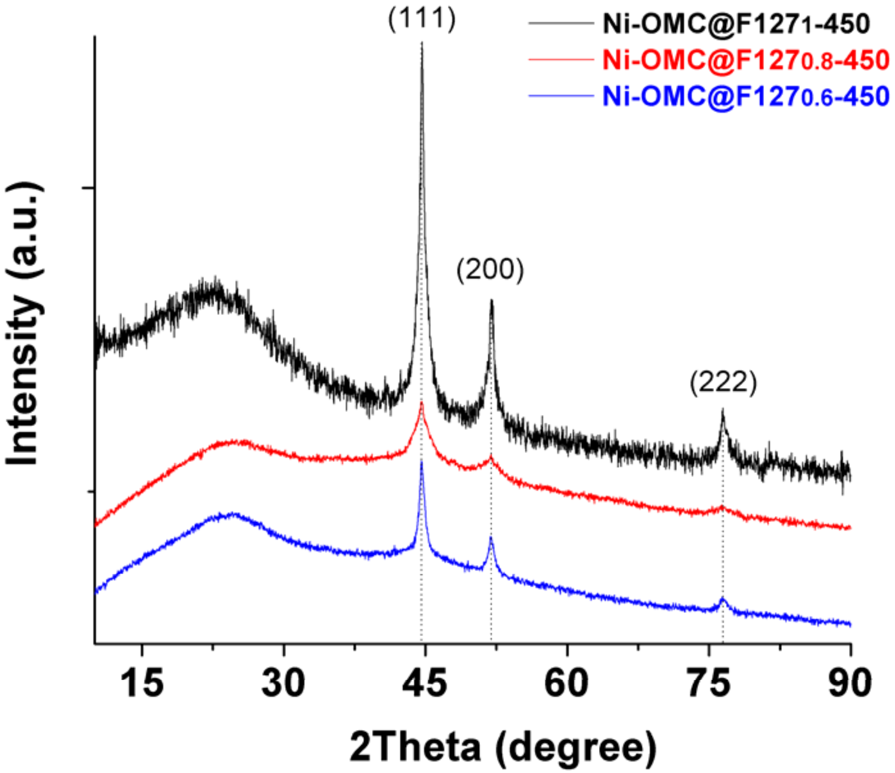

Supplementary Figure 22. XRD patterns of Ni-OMC@F127$_1$-450, Ni-OMC@F127$_{0.8}$-450 and Ni-OMC@F127$_{0.6}$-450. The average crystalline size by Scherrer's equation are 17.3, 5.7 and 12.3 nm for Ni-OMC@F127$_1$-450, Ni-OMC@F127$_{0.8}$-450 and Ni-OMC@F127$_{0.6}$-450 samples. Patterns were vertically offset for clarity

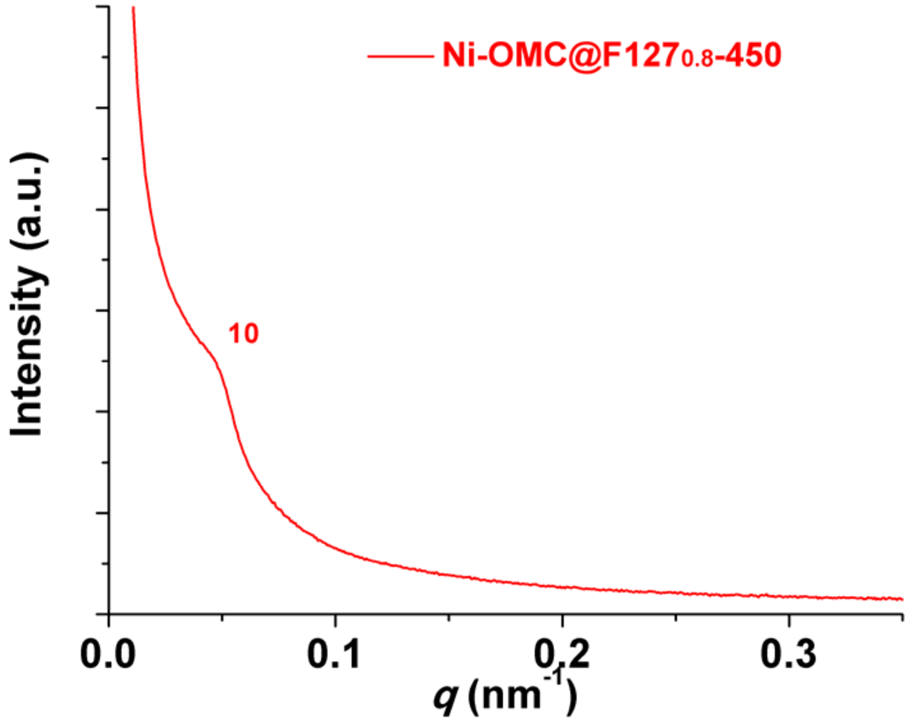

Supplementary Figure 23. SAXS pattern of Ni-OMC@$F127_{0.8}$-450 sample.

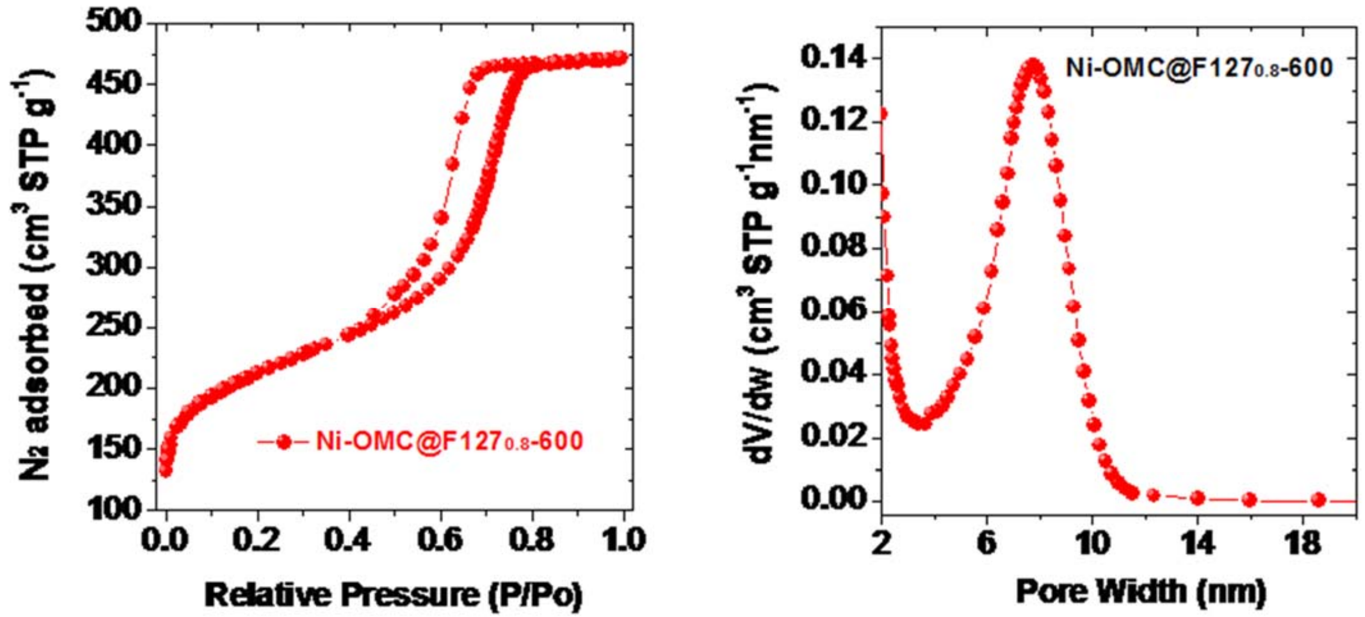

Supplementary Figure 24. 77 K nitrogen adsorption-desorption isotherm of Ni-OMC@F127$_{0.8}$-600 sample.

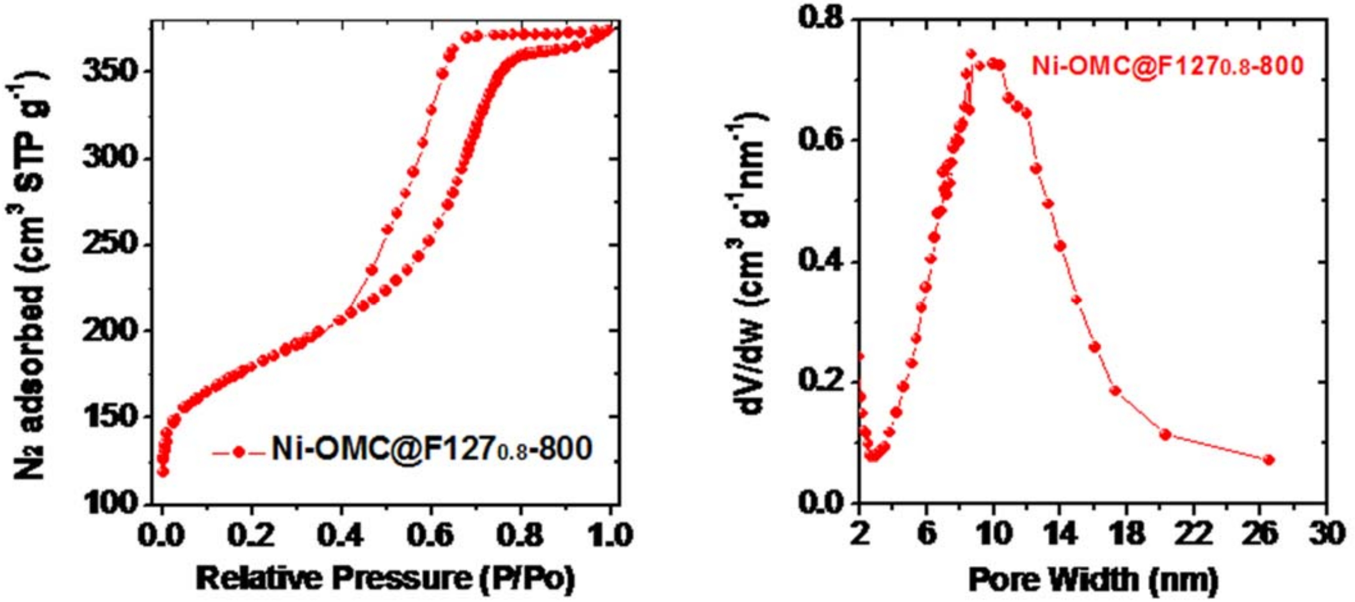

Supplementary Figure 25. The nitrogen adsorption-desorption isotherm (77 K) and pore size distribution of Ni-OMC@F127$_{0.8}$-800 sample.

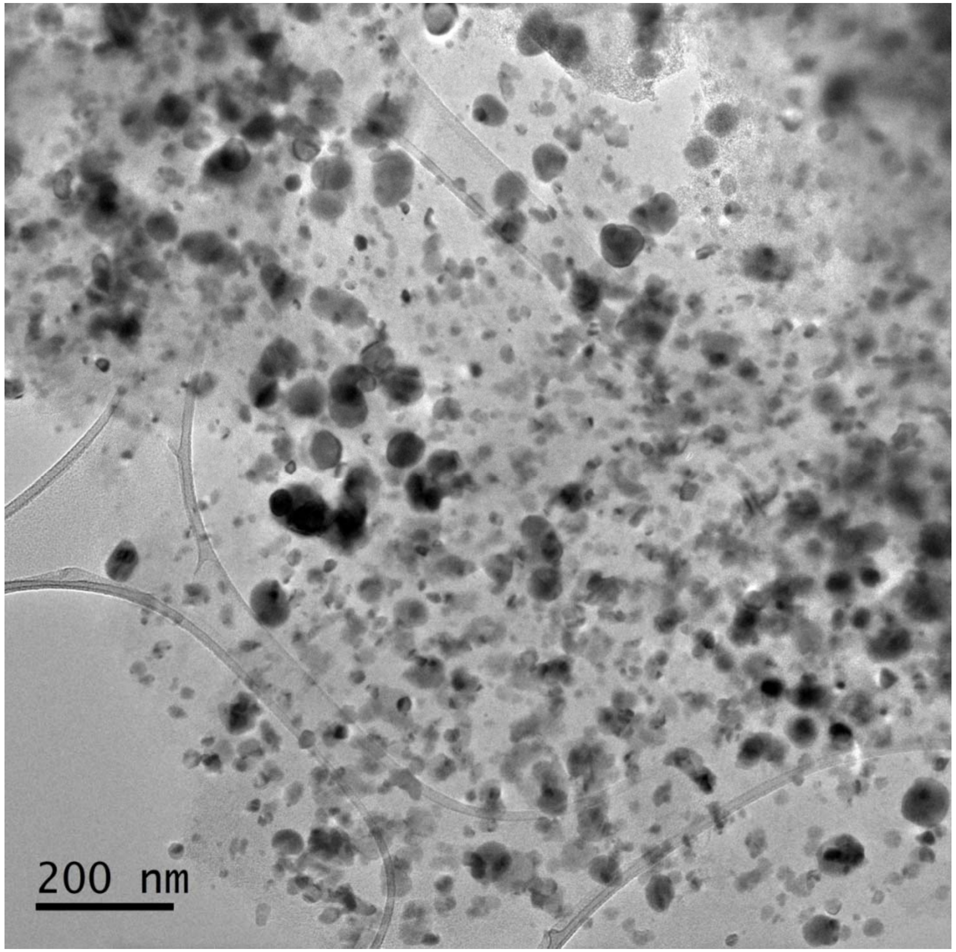

Supplementary Figure 26. TEM image of Ni-AC@450, a control sample with Ni supported on activated carbon by thermal treatment at 450°C. The Ni NPs aggregate critically with many Ni particles even bigger than 50 nm.

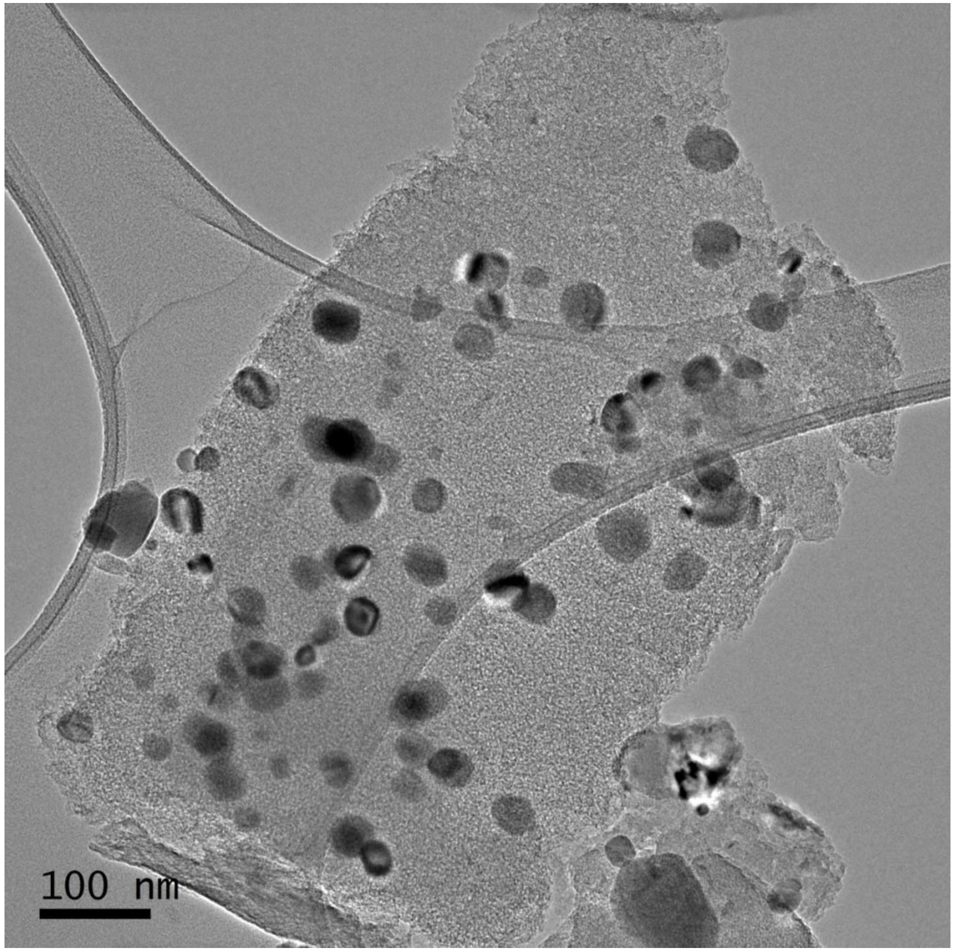

Supplementary Figure 27. TEM image of Ni-AC@800, a control sample with Ni supported on activated carbon by thermal treatment at 800°C. A serious growth of Ni NPs occurs. All Ni particles are bigger than 50 nm, and some are even bigger than 100 nm.

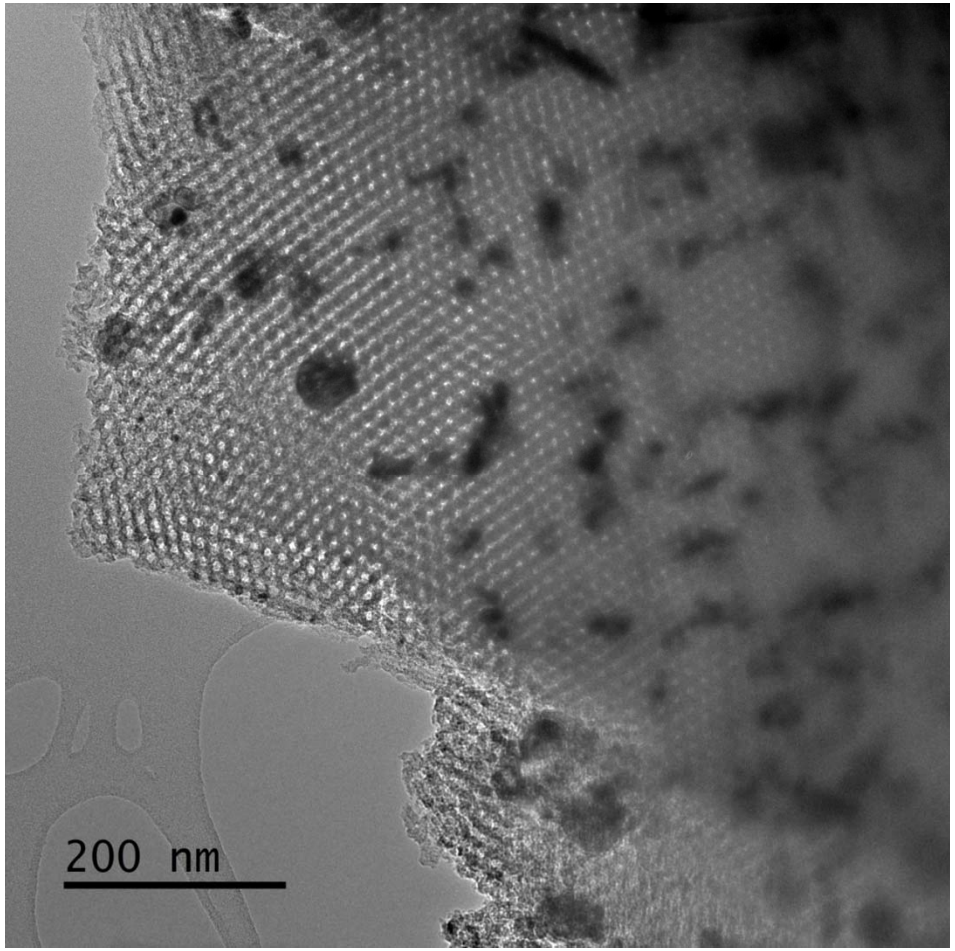

Supplementary Figure 28. TEM image of Ni-ST-OMC@450, a control sample with Ni supported on traditional soft-templating carbon by thermal treatment at 450°C. Some Ni particles are located in mesoporous channels; however, many Ni particles have already grown to a big size above 50 nm.

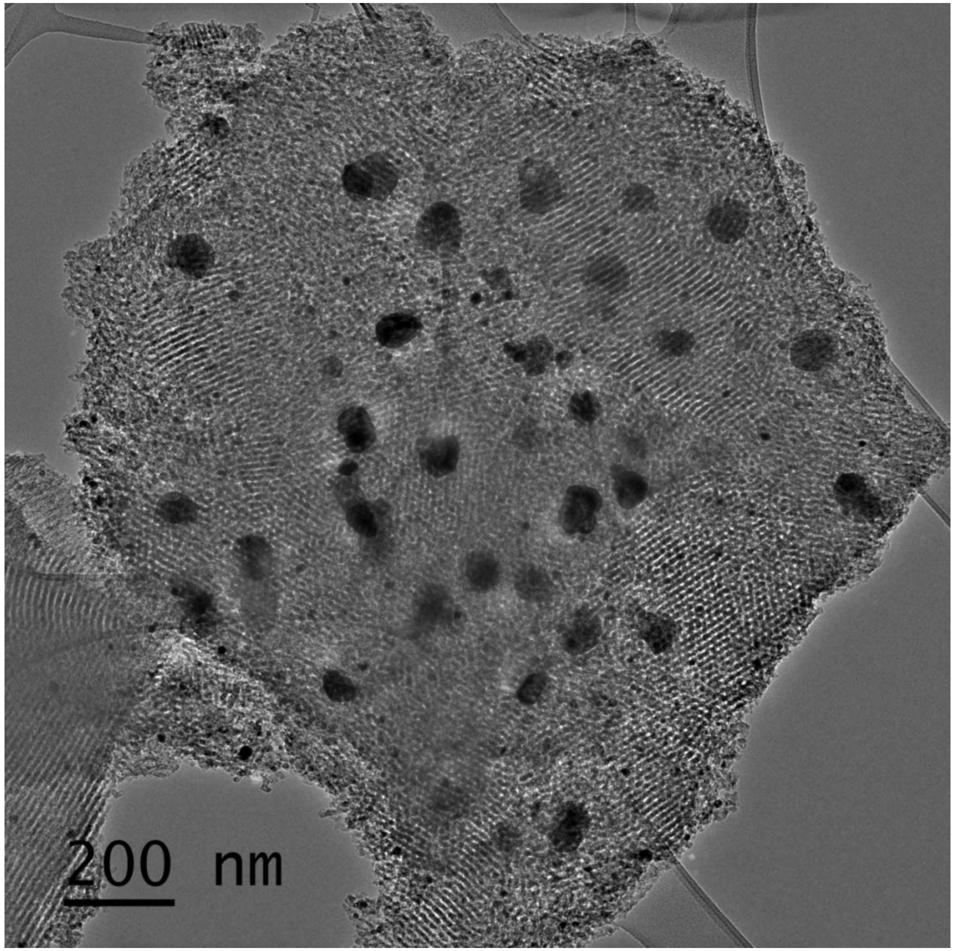

Supplementary Figure 29. TEM image of Ni-ST-OMC@800, a control sample with Ni supported on traditional soft-templating carbon by 800°C treatment.

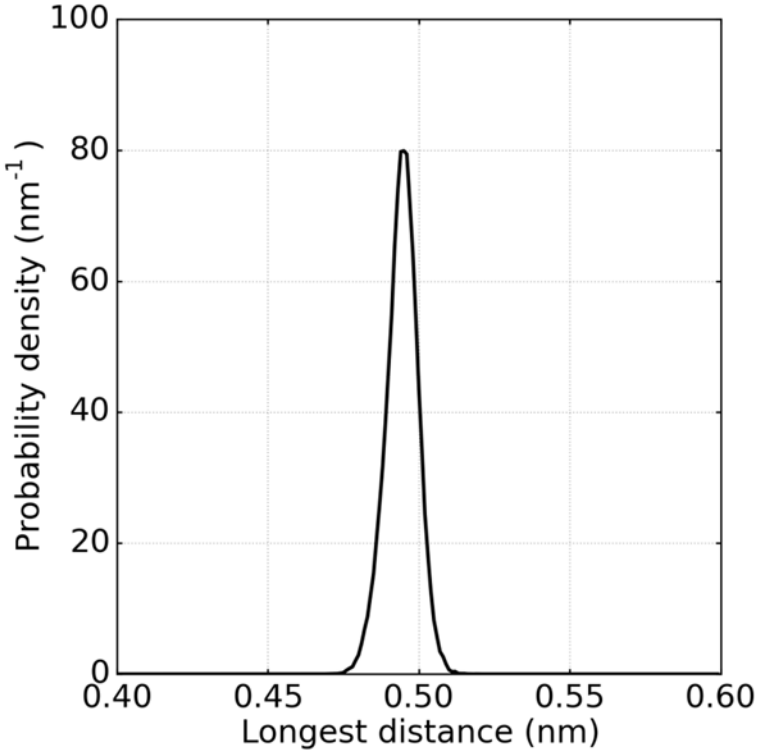

Supplementary Figure 30. A MD simulation of cyclohexene in vacuum.

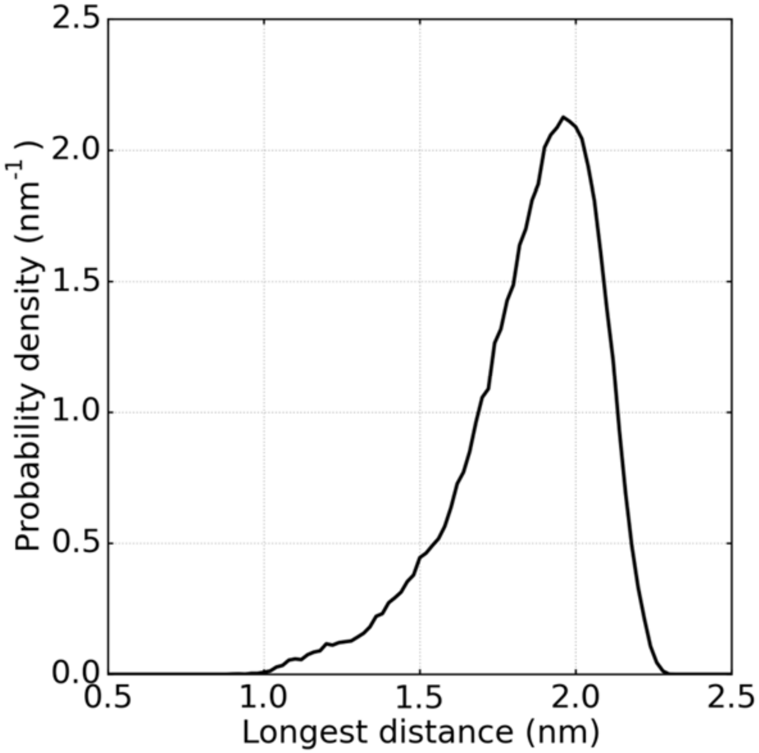

Supplementary Figure 31. A MD simulation of 1-octadecene in vacuum.

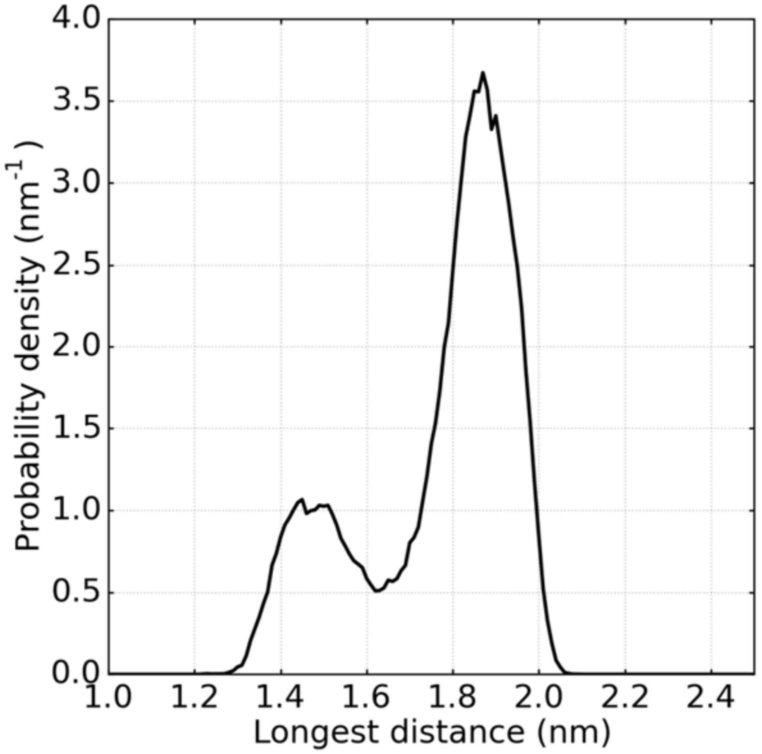

Supplementary Figure 32. A MD simulation of cholesteryl acetate in vacuum.

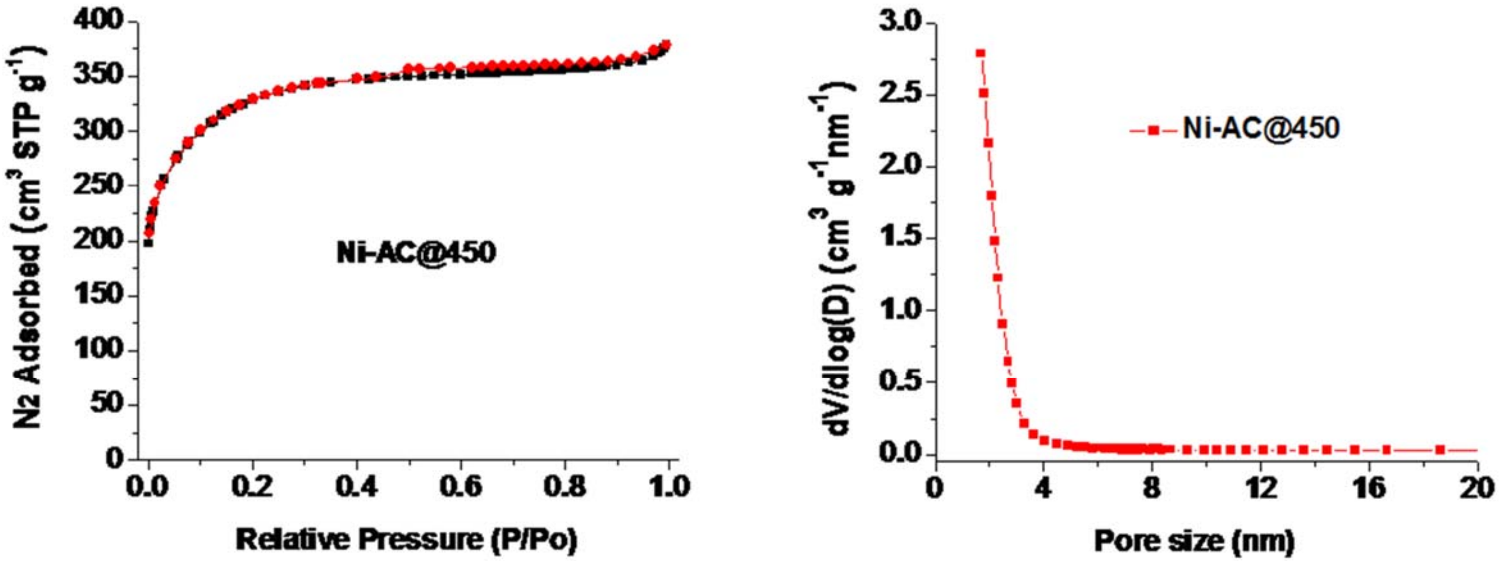

Supplementary Figure 33. The N_2 adsorption-desorption isotherm (77 K) and the corresponding pore size distribution of Ni-AC@450. The BET specific surface area is 1154 m^2g^{-1}.

Supplementary Table 1. The relationship between pore size and F-series surfactants used in the assembly.

	F38	F68	F87	F88	F127
Molecular Weight of PPO Chain	950	1750	2250	2250	4000
Pore Size (nm)	5.3	5.3	5.9	5.7	6.9

Supplementary Table 2. Selective Hydrogenation of Different Molecules by Ni Catalysts. [a]

Entry	Substrate	Product	Time (h)	Conv.	Sel.
1	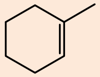	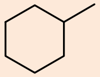	2	57	>99
2	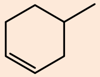	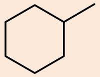	2	91	>99
3	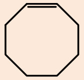	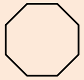	2.5	97	>99
4	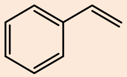	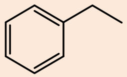	1.5	>99	>99

[a] Reaction Condition: substrate 1 mmol, ethanol 3 mL, Ni-OMC@F127$_{0.8}$-450 catalyst 10 mg, H_2 3 Mpa.

Supplementary Table 3. Selective hydrogenation of cyclohexene by recycled Ni catalyst. [a]

Run	Fresh	1st Reused	2nd Reused	3rd Reused	4th Reused	5th Reused
Yield	98	98	95	95	97	95

[a] Reaction Condition: cyclohexene 1 mmol, ethanol 3 mL, Ni-OMC@F127$_{0.8}$-450 catalyst (fresh or recycled), H_2 3 Mpa, 2 h.

Supplementary Table 4. A brief comparison of different methods for OMCs.

	Soft Template Method	Our Method
Reaction	Organic Poly-Condensation	Coordination Polymerization
Process	Solution-based<br>Processing for Membranes	Solvent-free Mechanochemistry
Precursor	Phenol-Aldehyde	Biomass-derived Tannin
Time	1-3 days	1 hour
Product	Pure OMCs	Pure OMCs or Metal-OMCs

Supplementary Note 1:

Simulation setup: Molecular dynamics simulations of the reactants in the vacuum were performed using MD package GROMACS[1] in the NVT ensemble. The temperature of was maintained at 298 K using a Nosé–Hoover thermostat[2]. The OPLS-AA[3, 4] force field was adopted for the reactants in this study. Each molecule was put in a cubic simulation box, and the initial configuration was generated by PRODRG[5]. Periodic boundary conditions were applied in all three directions. The electrostatic interactions were computed using the particle mesh Ewald method[6] with a 1.1 nm cutoff distance for real space. The van der Waals interaction was computed by direct summation with a cutoff distance of 1.1 nm. The LINCS algorithm[7] was used to maintain the bond lengths in each reactant. The system was first equilibrated for 5 ns, and then followed by a production run of 5 ns. A 1 fs time step was used and the trajectories were saved every 100 fs. For each frame of the trajectory, the maximum distance between 2 atoms was calculated. The distribution of the longest distance of the molecule over the entire trajectory was hence obtained by using the method of histogram.

Supplementary References

1. Hess, B., Kutzner, C., Van Der Spoel, D. &Lindahl, E. GRGMACS 4: Algorithms for highly efficient, load-balanced, and scalable molecular simulation. *J. Chem. Theory Comput.* **4,** 435–447 (2008).

2. Hoover, W. G. Canonical dynamics: Equilibrium phase-space distributions. *Phys. Rev. A* **31,** 1695–1697 (1985).

3. Jorgensen, W. L., Maxwell, D. S. & Tirado-Rives, J. Development and testing of the OPLS all-atom force field on conformational energetics and properties of organic liquids. *J. Am. Chem. Soc.***118,** 11225–11236 (1996).

4. Price, M. L. P., Ostrovsky, D. & Jorgensen, W. L. Gas-phase and liquid-state properties of esters, nitriles, and nitro compounds with the OPLS-AA force field. *J. Comput. Chem.* **22,** 1340–1352 (2001).

5. Schüttelkopf, A. W. & Van Aalten, D. M. F. PRODRG: A tool for high-throughput crystallography of protein-ligand complexes. *ActaCrystallogr. Sect. D Biol. Crystallogr.* **60,** 1355–1363 (2004).

6. Essmann, U. *et al.* A smooth particle mesh Ewald method. *J Chem Phys* **103,** 8577–8593 (1995).

7. Hess, B., Bekker, H., Berendsen, H. J. C. &Fraaije, J. G. E. M. LINCS: A linear constraint solver for molecular simulations. *J. Comput. Chem.* **18,** 1463–1472 (1997).
